# Synthesis, Characterization and Assessment of Antioxidant and Melanogenic Inhibitory Properties of Edaravone Derivatives

**DOI:** 10.3390/antiox13091148

**Published:** 2024-09-23

**Authors:** R. Divya Mohan, S. A. Anaswara, Naveen V. Kulkarni, Dimitar G. Bojilov, Stanimir P. Manolov, Iliyan I. Ivanov, Jamelah S. Al-Otaibi, Y. Sheena Mary

**Affiliations:** 1 Department of Chemistry, Amrita Vishwa Vidyapeetham, Amritapuri 690525, India; divyamohanr@am.amrita.edu (R.D.M.); anaswaraplr@gmail.com (S.A.A.); 2 Department of Organic Chemistry, University of Plovdiv, 24 Tzar Assen str., 4000 Plovdiv, Bulgaria; bozhilov@uni-plovdiv.net (D.G.B.); iiiliyan@abv.bg (I.I.I.); 3 Department of Chemistry, College of Science, Princess Nourah bint Abdulrahman University, P.O. Box 84428, Riyadh 11671, Saudi Arabia; dr.jamelah@gmail.com; 4 Department of Physics, FMNC, University of Kerala, Kollam 691001, India; sheena@fatimacollege.net

**Keywords:** edaravone derivatives, copper(II) complexes, antioxidant activity, molecular docking, molecular dynamics simulation, tyrosinase-binding, melanogenic inhibition activity

## Abstract

A series of edaravone derivatives and the corresponding Cu(II) complexes were synthesized and characterized using spectroscopic and analytical techniques such as IR, UV, NMR and elemental analysis. Antioxidant activities of all compounds were examined using free radical scavenging methods such as hydrogen peroxide scavenging activity (HPSA), 1,1-diphenyl-2-picrylhydrazyl (DPPH) and 2-2′-azino-bis-(3-ethylbenzothiazoline-6-sulfonate) (ABTS) assays. All of the tested compounds exhibited good antioxidant activity. Further, the frontier orbital energy levels, as well as various chemical properties, were determined using the density functional theory (DFT) calculations. The MEP maps of all of the derivatives were plotted to identify the nucleophilic and electrophilic reactive sites. Further, binding energies of all of the organic compounds with the protein tyrosinase was investigated to determine their potential anti-melanogenic applications. The selected ligand, **L6** was subjected to molecular dynamics simulation analysis to determine the stability of the ligand–protein complex. The MD simulation was performed (150 ns) to estimate the stability of the tyrosinase–**L6** complex. Other key parameters, such as, RMSD, RMSF, Rg, hydrogen bonds, SASA and MMPBSA were also analyzed to understand the interaction of **L6** with the tyrosinase protein.

## 1. Introduction

Edaravone is also known as methylphenylpyrazolone or norphenazone. It functions as a free radical scavenger and its metabolites offer a range of medicinal benefits, including antibacterial, anticancer activity, and protection against dementia caused by insufficient sleep. Its other bioactivities include preventing ferroptosis in patients with amyotrophic lateral sclerosis (ALS), treating acute pancreatitis, craniocerebral injuries, acute ischemic stroke, Alzheimer’s and Parkinson diseases, reducing oxygen species, and treating asthma through anti-inflammatory and anti-oxidative effects [1,2,3,4,5,6,7,8,9]. Its therapeutic impact may be attributed to its antioxidant qualities, though its exact method of action is not well understood [10,11]. Edaravone is proposed to have three main different mechanisms against oxidative stress. It operates by preventing the peroxyl radical-induced peroxidation of both the lipid and water-soluble systems, by stopping the lipoxygenase and non-enzymatic peroxidation of lipids or by quenching the hydroxyl radicals (OH•) and preventing the peroxidation of lipids [11]. Despite these promising antioxidant properties, oral bioavailability of the edaravone is restricted owing to its low stability in physiological conditions and poor solubility. Hence, oral formulations of edaravone have not yet been applied in a clinical setting [12,13]. It is evident that the electron density of the pyrazolone ring greatly influences the electron transfer mechanism and thus governs the antioxidant properties [13]. Introduction of the substituents with appropriate stereo-electronic properties could lead to the enhancement of the antioxidant properties of the edaravone and aid in the improvement of lipophilicity [14,15,16,17,18,19]. Derivatization of the edaravone can be achieved by four key routes: by substituting the N-phenyl ring, by modifying/derivatizing ketonic group (5-position), by changing the methyl group (3-position) or by substituting the pyrazole ring (4-position) [20,21,22,23,24,25,26,27,28]. All of these derivatization strategies have been found to be successful in achieving the obtained bio-reactivities; most particularly, the substitution of the various groups at the 4th position of edaravone ring has been shown to lead to the formation of compounds with high lipophilicity and high potential antioxidant properties [17,18,19].

The main factor determining skin tone in humans is the production of melanin, which also helps to shield the skin from ultraviolet radiation. However, excessive melanin production and aggregation can cause wrinkles, melisma, freckles, actinic damage sites, and other hyperpigmentation issues [29]. Melanogenic antagonists are becoming increasingly crucial components in drugs and cosmetics to stop hyperpigmentation [30,31,32]. During the initial phases of melanin production, o-diphenols and o-diquinones are oxidized by an enzyme called tyrosinase, which contains a copper center. This enzyme is also the reason for the undesired browning of damaged fruits [33,34,35]. Earlier studies on human skin cells have demonstrated that photodamage results in the production of reactive oxygen species (ROS) and a breakdown of endogenous antioxidant pathways. Additionally, it has been established that ROS are important regulators of melanocyte growth and melanin production, and that ROS scavengers and inhibitors can reduce UV-induced melanogenesis and hyperpigmentation [35,36,37].

Here, we report the synthesis, characterization, and evaluation of antioxidant activities of edaravone derivatives. Derivatization of the edaravone was undertaken by introducing different motifs of a versatile nature at the 4th position of the pyrazolone ring [20,21,22,23,24,25,26,27,28]. All of the synthesized organic compounds were complexed with copper in anticipation of further enhancement in the antioxidant properties [38,39,40,41]. After thorough characterization, all of the compounds were evaluated for their antioxidant potency by using free radical scavenging assays, such as hydrogen peroxide scavenging activity (HPSA), 1,1-diphenyl-2-picrylhydrazyl (DPPH) and 2-2′-azino-bis-(3-ethylbenzothiazoline-6-sulfonic acid) (ABTS) assays. DFT analysis of the organic compounds was undertaken to determine the frontier molecular orbital energy levels and theoretical chemical characteristics. The binding energies of the organic compounds with the protein tyrosinase, which plays a key role in melanogenesis [35,36,37], were analyzed with the help of docking studies. Molecular dynamics simulation studies were used to understand the interaction of a selected compound with tyrosinase.

## 2. Experimental

### 2.1. Materials and Methods

All of the chemicals used in the synthesis work, including organic precursors, reagents, metal salts and solvents were obtained from Sigma Aldrich (Bengaluru, India), Spectrochem or Nice Chemicals and were used after the appropriate purification and drying [42]. Molar conductivity of the complexes was measured using an ELICO–CM-82 conductivity bridge. Infrared spectral analysis of the organic compounds and metal complexes was undertaken on a Perkin Elmer Spectrum-2 FT-IR instrument (Waltham, USA). Electronic spectral analysis (UV–visible spectral studies) was performed using a Perkin Elmer LAMDA-25 instrument (Waltham, USA). ^1^H and ^13^C NMR analysis of the organic compounds was carried out on a Bruker Avance 400 MHz spectrometer. Elemental CHN analysis of all of the of the compounds was carried out using a Thermo Scientific Flash 2000 Organic Elemental Analyzer. Copper and chloride content of the complexes was analyzed by following the established gravimetric methods [43]. X-ray analysis of the single crystals was performed on a Bruker APEX-II Kappa machine.

In the antioxidant activity studies, HPLC grade methanol (VWR, Vienna, Austria) was used. Water for HPLC was prepared with a Millipore purifier (Millipore, St. Louis, MO, USA). Potassium dihydrogen phosphate, dipotassium hydrogen phosphate, hydrogen peroxide, ascorbic acid, 2,2-diphenyl-1-picrylhydrazyl (DPPH) and 2,2′-azino-bis(3-ethylbenzothiazoline-6-sulfonic acid) (ABTS) were purchased from Sigma Aldrich. A Camspec M508 spectrometer (Leeds, England) was used in the bioactivity assays.

### 2.2. Synthesis of Edaravone Derivatives

The core molecule, 5-methyl-2-phenyl-2,4-dihydro-3H-pyrazol-3-one (edaravone) (**L1**) was purchased from Sigma Aldrich and all of the derivatives were prepared by following the procedures described here below.

#### 2.2.1. 4-Acetyl-5-methyl-2-phenyl-2,4-dihydro-3H-pyrazol-3-one (L2)

**L2** was prepared by employing a known approach with some modifications. **L1** (7.5 g, 43 mmol) was taken in 35 mL tetrahydrofuran, to which Ca(OH)_2_ (6 g, 81 mmol) was added and then stirred. After 10 min, 5 mL acetyl chloride was added dropwise and stirring continued. The orange precipitate formed was refluxed for 4 h. The reaction mixture was cooled on an ice bath and 50 mL 2 M HCl was added with stirring until a cream-colored precipitate was separated. The precipitate was washed with excess water and dried. Yield—80%; melting point—135–137 °C; IR (KBr pellet, cm^−1^) 3333 (s, br), 3058 (m), 2933 (w), 2174 (w) 1633 (s), 1592 (s),1499 (s), 1400 (m), 1343 (m), 1260 (w), 1205 (w), 1157 (w), 1083 (s), 1022 (m), 963 (s), 841 (m), 752 (s), 687 (s), 597 (m); UV-vis (DMSO, 10^−4^ M, λ in nm (abs)) 265 (0.425), 288 (0.224); ^1^H NMR (400 MHz, DMSO-*d*_6_, ppm) 8.04 (2H, d (*J* = 8 Hz), o-*H*_Ph_), 7.29 (2H, t (*J* = 8 Hz), m-*H*_Ph_), 7.02 (1H, t (*J* = 8 Hz), o-*H*_Ph_), 2.28 (3H, s, (CO)C*H*_3_), 2.26 (3H, s, C*H_3_*_ring_); ^13^C NMR (100 MHz, DMSO-*d*_6_, ppm) 190.2, 165.1, 148.1, 140.4, 128.2, 122.8, 118.3, 104.3, 28.2, 17.3; analysis for C_12_H_12_N_2_O_2_ calc. (found) C 66.65 (66.24); H 5.59 (5.32), N 12.96 (12.88).

#### 2.2.2. 5-Methyl-2-phenyl-4-(1-(phenylimino)ethyl)-2,4-dihydro-3H-pyrazol-3-one (**L3**)

An amount of 1 g (4.6 mmol) **L2** was placed in 20 mL methanol, to which was added 0.49 mL (4.6 mmol) aniline. The mixture was refluxed overnight in the presence of a catalytic amount of acetic acid. The solid obtained was separated by filtration, thoroughly washed with MeOH and air-dried. Yield—82%; melting point—174–178 °C; IR (KBr pellet, cm^−1^) 3461 (m), 3124 (s, br), 2984 (m), 2527 (w) 1626 (s), 1580 (s), 1492 (m), 1482 (s), 1385 (s), 1224 (m), 1079 (s), 1042 (s), 1017 (s), 957 (s), 879 (m), 842 (s), 761 (s), 693 (s), 603 (s); UV–vis (DMSO, 10^−4^ M, λ in nm (abs)) 261 (0.443), 290 (0.224); ^1^H NMR (400 MHz, DMSO-d6, ppm) 12.97 (1H,s, O*H*), 8.02 (2H, d (*J* = 8 Hz), o-*H*_PhEda_), 7.52 (2H, t (*J* = 8 Hz), m-*H*_PhEda_), 7.40 (5H, m, *H*_Ph_), 7.14 (1H, t (*J* = 8 Hz), p-*H*_PhEda_), 2.45 (3H, s, (CO)C*H*_3_), 2.38 (3H, s, C*H_3_*_ring_); analysis for C_18_H_17_N_3_O calc. (found) C 74.20 (74.08), H 5.88 (6.02); N 14.42 (14.82).

#### 2.2.3. 5-Methyl-2-phenyl-4-(1-(2-phenylhydrazineylidene)ethyl)-2,4-dihydro-3H-pyrazol-3-one (**L4**)

An amount of 1 g (4.6 mmol) **L2** was placed in 20 mL methanol, to which was added 0.6652 g (4.6 mmol) phenyl hydrazine. The mixture was refluxed overnight in the presence of a catalytic amount of acetic acid. The solid obtained was separated by filtration, thoroughly washed with MeOH and air-dried. Yield—83%; melting point—186–188 °C; IR (KBr pellet, cm^−1^) 3462 (s,br), 3279 (s), 3058 (m), 1624 (s), 1538 (m), 1498 (s), 1434 (w), 1391 (m), 1309 (w), 1230 (w), 1147 (w), 1044 (w), 950 (w), 893 (w), 751 (m), 696 (m); UV–vis (DMSO, 10^−4^ M, λ in nm (abs)) 262 (0.799), 280 (0.895), 332 (0.462); ^1^H NMR (400 MHz, CDCl_3,_ ppm) 12.36 (1H,s, O*H*), 8.00 (2H, d (*J* = 8 Hz), o-*H*_PhEda_), 7.40 (2H, t (*J* = 8 Hz), m-*H*_PhEda_), 7.25 (2H, t (*J* = 8 Hz), m-*H*_Ph_), 7.17 (1H, t (*J* = 8 Hz), p-*H*_PhEda_), 6.95 (1H, t (*J* = 8 Hz), p-*H*_Ph_), 6.75 (2H, d (*J* = 8 Hz), o-*H*_Ph_), 6.17 (1H, s, N*H*), 2.45 (3H, s, (CO)C*H_3_*), 2.41 (3H, s, C*H_3_*_ring_); ^13^C NMR (100 MHz, CDCl_3_, ppm) 168.0, 165.5, 147.4, 146.3, 138.9, 129.5, 128.8, 124.5, 122.0, 119.3, 113.2, 98.8, 17.40, 14.26; analysis for C_18_H_18_N_4_O calc. (found) C 70.57 (70.81), H 5.92 (5.71), N 18.29 (18.98).

#### 2.2.4. 5-Methyl-2-phenyl-4-(1-(pyridin-2-ylimino)ethyl)-2,4-dihydro-3H-pyrazol-3-one (**L5**)

An amount of 1 g (4.6 mmol) **L2** was placed in 20 mL methanol, to which was added 0.435 g (4.6 mmol) 2-aminopyridine. The mixture was refluxed overnight in the presence of a catalytic amount of acetic acid. The solid obtained was separated by filtration, thoroughly washed with MeOH and air-dried. Yield—84%; melting point—250–252 °C; IR (KBr pellet, cm^−1^) 3448 (s,br), 1629 (s), 1595 (m), 1507(s), 1446 (w), 1358 (m), 1203 (w), 1085 (m), 1022 (w), 962 (s), 875 (w), 843 (m), 765 (s), 695 (m), 664 (w), 604 (w); UV–vis (DMSO, 10^−4^ M, λ in nm (abs)) 267 (0.483), 286 (0.331); ^1^H NMR (400 MHz, DMSO-*d*_6_, ppm) 12.20 (1H, s, O*H*), 8.74 (1H, s, o-*H*_Py_), 7.96 (2H, d (*J* = 8 Hz), o-*H*_PhEda_), 7.35 (2H, t (*J* = 8 Hz), m-*H*_PhEda_), 7.09 (1H, t (*J* = 8 Hz), p-*H*_PhEda_), 6.42 (3H, b, p-*H*_Py_), 2.34 (3H, s, (CO)C*H_3_*), 2.31 (3H, s, C*H_3_*_ring_); ^13^C NMR (100 MHz, DMSO-d6, ppm) 166.9, 165.2, 157.9, 147.7, 139.6, 129.1, 124.1, 118.3, 97.7, 17.3, 14.7; analysis for C_17_H_16_N_4_O calc. (found) C 69.85 (69.28), H 5.52 (5.23), N 19.17 (19.86).

#### 2.2.5. 2-(1-(3-Methyl-5-oxo-1-phenyl-4,5-dihydro-1H-pyrazol-4-yl)ethylidene)hydrazine-1-carboxamide (L6)

An amount of 1 g (4.6 mmol) **L2** was placed in 20 mL methanol, to which was added 0.516 g semicarbazide. The mixture was refluxed overnight in the presence of a catalytic amount of acetic acid. The solid obtained was separated by filtration, thoroughly washed with MeOH and air-dried. Yield—72%; melting point—214–217 °C; IR (KBr pellet, cm^−1^) 3399 (s), 3181 (s), 3039 (s), 2929 (s), 1953 (w), 1710 (s), 1631 (s), 1540 (m), 1485 (s), 1452 (s), 1361 (s), 1211 (w), 1107 (w), 1018 (m), 915 (m), 751 (s), 695 (s), 653 (m), 609 (m); UV–vis (DMSO, 10^−4^ M, λ in nm (abs)) 271 (0.572); analysis for C_13_H_15_N_5_O_2_ calc. (found) C 57.13 (56.88), H 5.53 (5.68), N 25.63 (26.13); mass analysis: *m*/*z* 272 (M−H)^−^.

#### 2.2.6. 4-(1-(Hydroxyimino)ethyl)-5-methyl-2-phenyl-2,4-dihydro-3H-pyrazol-3-one (**L7**)

An amount of 1 g (4.6 mmol) of **L2** was placed in 20 mL methanol, to which was added 0.3217 g (4.6 mmol) hydroxyl amine hydrochloride. The mixture was refluxed overnight. The solid obtained was separated by filtration, thoroughly washed with MeOH and air-dried. Yield—76%; melting point—128–130 °C; IR (KBr pellet, cm^−1^) 3396(s, br), 2520 (w), 1963 (w), 1638 (s), 1516 (s), 1201 (w), 1081 (s), 1016 (s), 964 (s), 873 (m), 764 (s), 692 (s), 604 (s); UV–vis (DMSO, 10^−4^ M, λ in nm (abs)) 261 (0.386), 305 (0.173); analysis for C_12_H_13_N_3_O_2_ calc. (found) C 62.33 (62.87), H 5.67 (5.72), N 18.17 (18.64); mass analysis: *m*/*z* 230 (M−H)^−^.

#### 2.2.7. 5-Methyl-2-phenyl-4-thiocyanato-2,4-dihydro-3H-pyrazol-3-one (**L8**)

A mixture of **L1** (10 mmol, 1.74 g), ammonium thiocyanate (30 mmol, 2.28 g) and potassium persulphate (20 mml, 5.4 g) was placed in 30 mL acetonitrile in a 100 mL RB flask and stirred overnight. The yellow-orange colored product that formed was filtered and dried. Yield—80%; melting point—184–186 °C; IR (KBr pellet, cm^−1^) 3431 (br, m), 3160 (s), 1651 (w), 1404 (s), 1200 (m), 1115 (s), 623 (s); UV–vis (DMSO, 10^−4^ M, λ in nm (abs)) 265 (0.537), 425 (0.277); ^1^H NMR (400 MHz, CDCl_3_, ppm) 7.84 (2H, d (*J* = 8 Hz), o-*H*_PhEda_), 7.44 (2H, t (*J* = 8 Hz), m-*H*_PhEda_), 7.24 (1H, t (*J* = 8 Hz), p-*H*_PhEda_), 2.67 (3H, s, C*H_3_*_ring_); ^13^C NMR (100 MHz, CDCl_3_, ppm) 162.0, 148.7, 137.0, 133.6, 129.1, 128.9, 125.7, 119.1, 118.8, 19.4; analysis for C_11_H_9_N_3_OS calc. (found) C 57.13 (57.43), H 3.92 (3.74), N 18.17 (18.73).

#### 2.2.8. 3-Hydroxy-3-(3-methyl-5-oxo-1-phenyl-4,5-dihydro-1H-pyrazol-4-yl)indolin-2-one (**L9**)

A mixture of **L1** (1 g, 6 mmol) and isatin (0.883 g, 6 mmol) was refluxed at 80 °C in ethanol overnight. Upon completion of the reaction, the solid product was filtered, washed, and dried. Yield—75%; melting point—250–252 °C; IR (KBr pellet, cm^−1^) 3470 (br, s), 2830 (w), 2380 (w), 1740 (s), 1624 (s), 1500 (m), 1468 (m), 1409 (m), 1307 (m), 1235 (w), 835 (w), 757 (s), 693 (s); UV–vis (DMSO, 10^−4^ M, λ in nm (abs)) 263 (1.25), 347 (0.14); ^1^H NMR (400 MHz, CDCl_3_, ppm) 9.30 (1H, d (*J* = 4 Hz),*H*_Isa_), 7.93 (2H, d(*J* = 8 Hz), o-*H*_PhEda_), 7.61 (1H, s, -O*H_Isa_*) 7.44 (3H, m, pandm-*H*_PhEda_), 7.22 (1H, t (J = 8Hz) H_Isa_), 7.06 (1H, t (*J* = 8Hz) H_Isa_), 6.80 (1H, d (*J* = 8 Hz) H_Isa_), 2.64 (3H, s, C*H_3_*_ring_); ^13^C NMR (100 MHz, CDCl_3_, ppm) 167.3, 164.0, 148.7, 144.5, 138.9, 137.9, 132.2, 129.0, 119.2, 110.3, 20.0; analysis for C_18_H_15_N_3_O_3_ calc. (found) C 67.28 (67.54), H 4.71 (4.88), N 13.08 (13.52).

### 2.3. Synthesis of Complexes

All of the copper(II) complexes **C1**–**C9** were prepared by using the procedure detailed below. An amount of 10 mL methanolic solution of CuCl_2_.2H_2_O (1 mmol) was added dropwise to the 10 mL ligand solution (2 mmol, in methanol) with constant stirring. The mixture was heated at 60 °C overnight and the solid product obtained was separated by filtration, washed with methanol and hexane, and air-dried.

#### 2.3.1. Complex C1

Brown amorphous solid; yield—80%; melting point— >280 °C; IR (KBr pellet, cm^−1^) 591(w), 698 (m), 758 (s), 842 (w), 897 (w), 1034 (w), 1073 (w), 1169 (w), 1317 (w), 1370 (s), 1451 (s), 1491 (s), 1600 (s), 1883 (w), 1959 (w), 2345 (w), 3356 (s), 3444 (s), 3744 (w), 3841 (w), 3902 (w); UV–vis (DMSO, 10^−4^ M, λ in nm (abs)) 260 nm (0.23), 306 nm (0.066); analysis for C_20_H_24_Cl_2_CuN_4_O_4_ calc. (found) C 46.30 (46.73), H 4.66 (4.82), Cl 13.66 (14.08), Cu 12.25 (12.86), N 10.80 (11.32)%

#### 2.3.2. Complex C2

Green amorphous solid; yield—74%; melting point— >280 °C; IR (KBr pellet, cm^−1^) 3452 (s), 3340 (s), 3064 (m), 2969 (m), 2920 (m), 1835 (w), 1609 (s), 1593 (s), 1494 (s), 1380 (s), 1231 (m), 1089 (s), 1022 (m), 978 (s), 908 (m), 874 (m), 847 (m), 747 (s), 690 (s), 660 (m), 625 (s); UV–vis (DMSO, 10^−4^ M, λ in nm (abs)) 263 (0.459), 280 (0.413); analysis for C_24_H_24_Cl_2_CuN_4_O_4_ calc. (found) C 50.85 (51.21), H 4.27 (4.98), Cl 12.51 (12.31), Cu 11.21 (11.20), N, 9.88 (10.23)%

#### 2.3.3. Complex C3

Green amorphous solid; yield—82%; melting point— >280 °C; IR (KBr pellet, cm^−1^) 3456 (s, broad), 3066 (w), 2967 (w), 2923 (w), 2926 (w), 2345 (w), 1576(s), 1542(m),1496(s), 1437 (s), 1380 (s), 1089 (m), 1022 (m), 978 (w), 756 (w), 689 (w), 626(m), 497 (w); UV–vis (DMSO, 10^−4^ M, λ in nm (abs)) 264 (1.38), 551 (0.032); Analysis for C_36_H_34_C_l2_CuN_6_O_2_ calc. (found) C 60.29 (60.62), H 4.78 (5.02), Cl 9.89 (9.62), Cu 8.86 (8.43), N 11.72 (12.21)%

#### 2.3.4. Complex C4

Green amorphous solid; yield—76%; melting point— >280 °C; IR (KBr pellet, cm^−1^) 3400 (br, s), 3060 (m), 2919 (w), 1934 (w), 1616 (s), 1537 (s), 1484 (s), 1383 (s), 1232 (m), 1089 (s), 1028 (m), 978 (s), 907 (m), 848 (m), 747 (s), 689 (s), 661 (s), 625 (s); UV–vis (DMSO, 10^−4^ M, λ in nm (abs)) 262 (0.548), 282 (0.363); analysis for C_36_H_36_Cl_2_CuN_8_O_2_ calc. (found) C 57.87 (58.21), H 4.86 (4.92), Cl 9.49 (9.23), Cu 8.50 (8.12), N 15.00 (15.63)%

#### 2.3.5. Complex C5

Green amorphous solid; yield—70%; melting point— >280 °C; IR (KBr pellet, cm^−1^) 3447 (m), 3352 (m), 3065 (w), 2967 (w), 2928 (w), 1594 (s), 1541 (s),1499 (s), 1382 (m) 1088 (s), 1020 (m), 976 (w), 909 (w), 847 (w),755 (m), 690 (m), 625 (m), 496 (w); UV–vis (DMSO, 10^−4^ M, λ in nm (abs)) 260 (0.47), 283 (0.268); analysis for C_17_H_18_Cl_2_CuN_4_O_2_ calc. (found) C 45.91 (46.32), H 4.08 (4.28), Cl 15.94 (15.44), Cu 14.29 (13.84), N 12.60 (13.12)%

#### 2.3.6. Complex C6

Green amorphous solid; yield—85%; melting point— >280 °C; IR (KBr pellet, cm^−1^) 3912 (w), 3743 (w), 3603 (s), 3446 (s), 3396 (s), 3308 (s), 3174 (s), 1988 (w), 2359 (s), 1574 (s), 1491 (s), 1426 (s), 1350 (s), 1169 (s), 1061 (s), 987 (s), 903 (m), 855 (w), 758 (s), 663 (s), 585 (s), 501 (m), 451 (w); UV–vis (DMSO, 10^−4^ M, λ in nm (abs)) 269 (1.90), 325 (1.08); analysis for C_13_H_17_Cl_2_CuN_5_O_3_ calc. (found) C 36.67 (36.78), H 4.02 (3.92), Cl 16.65 (16.21), Cu 14.93 (15.23), N 16.45 (17.08)%

#### 2.3.7. Complex C7

Brown amorphous solid; yield—82%; melting point— >280 °C; IR (KBr pellet, cm^−1^) 3453 (s), 3343 (s), 3077 (w), 2968 (w), 2922 (w), 2340 (w), 1953 (w), 1731(w), 1610(s), 1514 (s), 1433 (s), 1027 (m), 949 (m),754 (m), 623 (w), 489 (w); UV–vis (DMSO, 10^−4^ M, λ in nm (abs)) 261 (0.471), 298 (0.179); analysis for C_24_H_26_Cl_2_CuN_6_O_4_ calc. (found) C 48.29 (48.72), H 4.39 (4.62), Cl 11.88 (12.14), Cu 10.65 (10.23), N 14.08 (14.43)%

#### 2.3.8. Complex C8

Brown amorphous solid; yield—76%; melting point— >280 °C; IR (KBr pellet, cm^−1^) 3458 (br, s), 3215 (br, s), 2254 (w), 2079 (w), 1635 (m), 1406 (s), 1121 (s), 980 (m), 768 (w), 615 (s); UV–vis (DMSO, 10^−4^ M, λ in nm (abs)) 275 (0.449), 415 (0.166); analysis for C_22_H_18_Cl_2_CuN_6_O_2_S_2_ calc. (found) C 44.26 (44.53), H 3.04 (2.81), Cl 11.88 (12.19), Cu 10.64 (10.31), N 14.08 (14.62)%

#### 2.3.9. Complex C9

Brown amorphous solid; yield—80%; melting point— >280 °C; IR (KBr pellet, cm^−1^) 3458 (s), 3365 (s), 2944 (w), 2376 (w), 1653 (s), 1504 (w), 1360 (w), 1356 (w), 991 (w), 852 (m), 766 (w), 613 (w), 515 (m); UV–vis (DMSO, 10^−4^ M, λ in nm (abs)) 317 (0.346), 360 (0.272), 490 (0.18); analysis for C_36_H_32_CuN_6_O_8_ calc. (found) C 58.41 (57.92), H, 4.36 (4.12), Cu 8.58 (9.12), N 11.35 (11.78)%

### 2.4. Evaluation of Antioxidant Properties

#### 2.4.1. Hydrogen Peroxide Scavenging Activity (HPSA) Assay Protocol

The hydrogen peroxide (H_2_O_2_) scavenging ability of all of the synthesized compounds was analyzed by following the reported method [44,45]. In a typical procedure, the test tubes were added with 0.6 mL of H_2_O_2_ (43 mM), 1 mL of the sample (variable concentration, 10–1000 μg/mL) and 2.4 mL of potassium phosphate buffer solution (0.2 M, pH 7.4). After thoroughly stirring, the mixture was incubated for ten minutes in the absence of light (at 37 °C). Change in the absorbance at λ_max_ = 230 nm was measured using a spectrophotometer. Ascorbic acid was used as standard. The percentage HPSA of the samples was calculated using the following formula:

$$I,\%(HPSA)=\left[\frac{A_{blank}-(A_{TS}-A_{CS})}{A_{blank}}\right]*100$$

where *A_blank_*, *A_cs_*, and *A_TS_* are the absorbance values of the blank sample (phosphate buffer + H_2_O_2_), the control sample (compound + phosphate buffer) and the test sample (compound + phosphate buffer + H_2_O_2_), respectively. The IC_50_ values were obtained by interpolating the graphical dependence of hydrogen peroxide on concentration. An average value of three experiments is reported.

#### 2.4.2. Free Radical Scavenging Assay

The DPPH (DPPH = 2,2-diphenyl-1-picrylhydrazyl) assay was carried out by following the reported procedure [46]. In a typical procedure, 2 mL of freshly prepared DPPH• solution was added with 2 mL of the test sample (various concentrations) and the mixtures were incubated for 30 min at RT. Change in the absorbance of the sample at λ_max_ = 517 nm was measured under low-light conditions. The percentage radical scavenging ability (RSA) of all of the compounds was evaluated by using the following formula:

$$RSA\;\%=\left[\frac{A0-Ab}{A0}\right]\times 100$$

where *A*0 and *Ab* are the absorbance values of the control and samples, respectively. The average IC_50_ value (of three sets) was calculated by the graphical method.

#### 2.4.3. ABTS (2,2′-Azino-bis(3-ethylbenzothiazoline-6-sulfonic acid) assay

The method reported by Re et al. was used for the ABTS assay [47]. 2,2′-azino-bis(3-ethylbenzothiazoline-6-sulfonic acid) cation radical (ABTS^•+^) solution was made by dissolving 7 mM of ABTS in 2.45 mM K_2_S_2_O_8_. The mixture was stirred for 16 h in the absence of light at RT. For the analysis, ABTS^•+^ stock solution was diluted with MeOH to obtain dilution with absorbance of 0.70 ± 0.02 (λ_max_ = 734 nm). In a typical analysis, 0.15 mL of compound solution (variable concentration) and 2.85 mL of ABTS solution were mixed and incubated at RT for 7 min, the change in the absorbance at λ_max_ = 734 nm was measured with the help of a spectrophotometer. The average IC_50_ values (set of three experiments) are determined as above.

#### 2.4.4. Statistical Analysis

All of the analyses were made in triplicates. Data were expressed as mean ± SD. The level of significance was set at *p* < 0.05. Statistical program SPSS 19.0 software was used for data analysis by ANOVA followed by Duncan’s post-hoc test to evaluate differences between mean values of activities (SPSS Inc., Chicago, IL, USA).

### 2.5. Computational Studies: Methadology

All calculations were undertaken with the DFT using Gaussian 16 and GaussView 6 software [48,49]. For all calculations, the B3LYP technique with the 6-311++G* basis set was utilized [50]. Calculations and comparisons were also undertaken for the chemical descriptors. Autodock-Vina achieved successful molecular docking [51].

From our experimental studies, the compound **L6** shows maximum biological activities and hence all molecules and metal complexes are docked and the binding affinities are found. In addition, molecular dynamics (MD) simulation of **L6** is also reported to validate the experimental results of L6 with the tyrosinase from *Bacillus megaterium* (PDB ID: 3NM8). To obtain a clean protein structure, water and heteroatoms were removed from 3NM8 [52]. Blind docking calculations for ligand molecules bound in interactions consider molecular binding sites. Autodock-Vina was used for the study of receptor–ligand docking, and the box size was 60 × 60 × 60 Å.

MD simulation was performed on the chosen protein–ligand complex in Gromacs-2019.4. To obtain the force field coordinates, the chosen ligand topology was obtained from PRODRG server [53] and MD simulations were performed as reported earlier using GROMACS suite to find different parameters and trajectory analysis [54,55].

To comprehend the binding free energy (ΔG binding) during the simulation, MM-PBSA was used using the function g_mmpbsa. We computed ΔG for the last 50 ns using dt 1000 frames to obtain an accurate result [56].

## 3. Results and Discussion

### 3.1. Chemistry

#### 3.1.1. Synthesis

Compound **L1** (edaravone) was obtained commercially and used without further purification. Compound **L2** was obtained by the acetylation of **L1** with acetyl chloride under alkaline reaction conditions [57]. Compounds **L3**–**L7** were obtained by reacting **L2** with stochiometric amounts of aniline, phenylhydrazine, 2-aminopyridine, semicarbazide and hydroxylamine in methanol, under mild acidic and reflux conditions [58]. Compound **L8** was obtained by reacting **L1** with ammonium thiocyanate in the presence of potassium persulphate in acetonitrile [59]. The last compound, **L9** was obtained by reacting **L1** with isatin under alkaline conditions [60].

**Figure 1:**
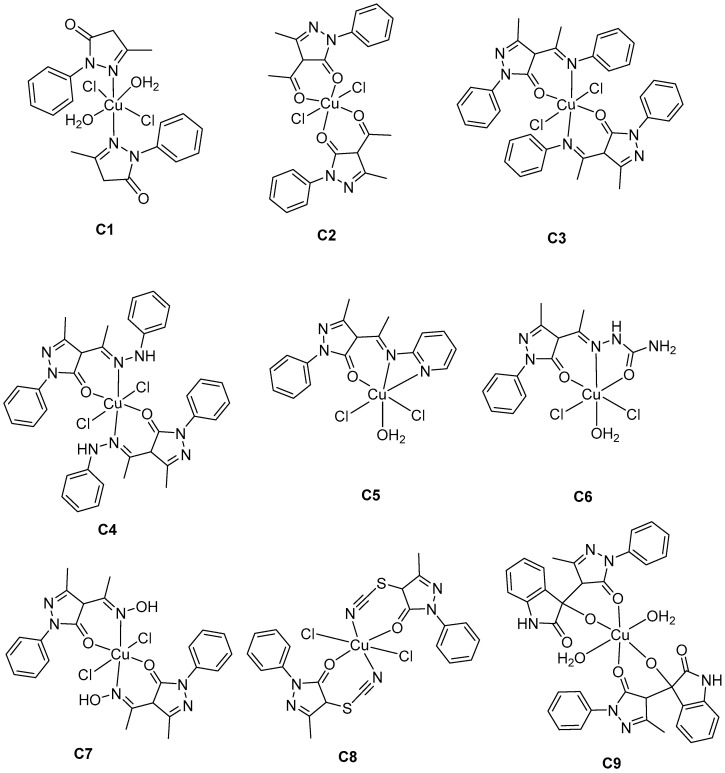
Proposed structures of copper complexes **C1**–**C9** (based on the spectral and analytical characterization).

The nine compounds, **L1**–**L9**, were treated with CuCl_2_.2H_2_O (in 1:1 or 1:2 stoichiometry) in methanol under reflux conditions to obtain the respective copper complexes, **C1**–**C9** in high yield (Figure 1).

#### 3.1.2. Characterization

All of the organic compounds, **L1**–**L9,** and their respective copper complexes, **C1**–**C9,** were duly characterized by using various spectro-analytical methods.

##### Elemental Analysis

**Scheme 1:**
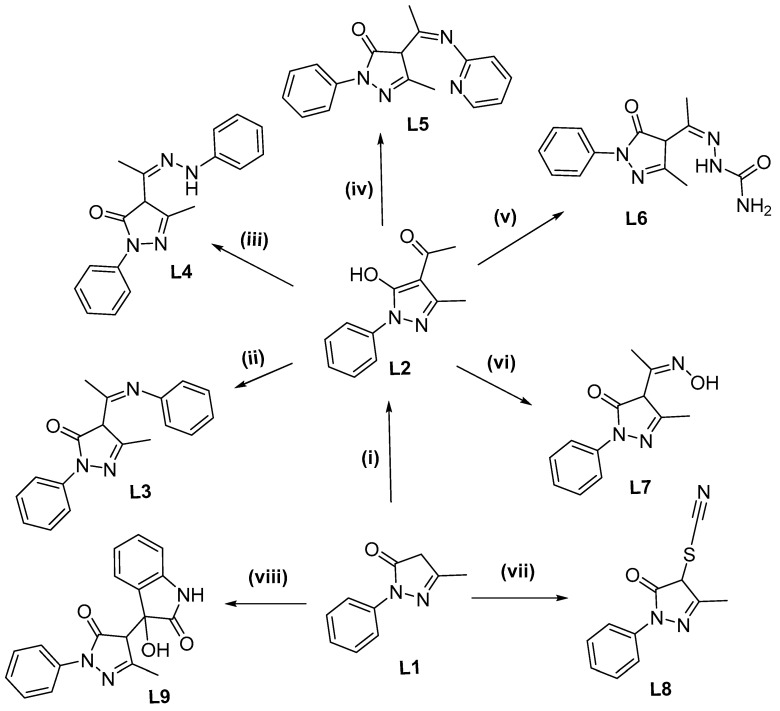
Synthetic route of compounds **L2**–**L9**. Reaction conditions (i) AcCl, Ca(OH)_2,_ THF, 4 h, reflux; (ii) aniline, MeOH, H^+^, 12 h, reflux; (iii) phenyl hydrazine, MeOH, H^+^, 12 h, reflux; (iv) 2-aminopyridine, MeOH, H^+^, 12 h, reflux; (v) semicarbazide, MeOH, H^+^, 12 h, reflux; (vi) hydroxylamine, MeOH, H^+^, 12 h, reflux; (vii) NH_4_SCN, K_2_S_2_O_8_, MeOH, 12 h, RT; and (viii) Isatin, NaOH, MeOH, 12 h, reflux.

The carbon, hydrogen and nitrogen analysis data obtained for all of the compounds fit well with the proposed structures of the ligands (Scheme 1) and complexes (Figure 1), hence confirming their analytical purity. The copper and chloride analysis data [43] of the complexes confirmed the projected 2:1 ligand to metal stochiometric ratio in the complexes, **C1**–**C4** and **C7**–**C9**, while 1:1 types of complex formation were found in the cases of **C5** and **C6**.

##### Infrared Spectral Analysis

The infrared spectral analysis provided valuable information about the structural features of the compounds. The important absorption bands of all of the compounds are provided in the experimental section (*vide infra*) and the corresponding spectra are provided in the Supplementary Materials. The medium/strong intensity broadbands observed in the range 3100–3500 cm^−1^ are ascribed to the stretching of -NH/-OH bonds in the ligands. These bands obtain breadth and intensity in the corresponding complexes owing to the coordination and presence of ligated/solvated water molecules. The medium intensity signals seen in the range 2900–3000 cm^−1^ in **L1**–**L9** are assigned to the C-H stretching vibrations. Ketonic, acetyl or azomethine absorptions are observed in the range 1690–1550 cm^−1^ in the ligands and experience considerable change in the corresponding complexes (**C1**–**C9**), indicating the metal coordination. The characteristic fingerprint region (1500–550 cm^−1^) of the ligand systems [57,58,59,60] experienced distinct changes upon complexation [61,62,63,64,65,66].

##### UV–Visible Spectral Analysis

UV–visible spectra of all of the organic derivatives (**L1**–**L9**) and copper complexes (**C1**–**C9**) were recorded in the range 250–900 nm in DMSO at 10^−4^ M concentration. All of the organic ligands exhibited broad peaks in the region 260–280 nm corresponding to π–π* transitions. Low energy transitions observed in the 280–300 nm and 350–450 nm regions are ascribed to the n-π* transitions associated with aromatic rings and N, O and S, heteroatoms present in the ligands. These peaks experience noticeable changes in the respective copper complexes owing to metal coordination [61,62,63,64,65,66]. The ligand-to-metal charge transfer (LMCT) transitions, which appear in the range 350–450 nm in some complexes, significantly increase the intensity of the bands in the region. A peak corresponding to the d–d transition was observed only in the complex **C3** (i.e., 550 nm). All of the absorption values of each compound are listed in the experimental section (*vide infra*) and the corresponding spectra are provided in the Supplementary Materials.

##### NMR Analysis

All of the synthesized edaravone derivatives (**L2**–**L9**) were duly characterized by ^1^H and ^13^C NMR spectral analysis (in CDCl_3_ or DMSO-*d*_6_ solvent) and compared with the related compounds reported in [57,58,59,60] (except for the compounds **L6** and **L7,** due to their low solubility). The peak assignment for each compound is depicted in the experimental section (vide infra) and the corresponding NMR spectra are provided in the Supplementary Materials. The NMR analysis of the respective copper (II) complexes was not completed owing to their paramagnetic nature.

##### Mass Analysis

ESI mass analyses of ligands **L6** and **L7** were carried out in order to confirm their molecular structures. The high intensity base peaks observed at *m*/*z* 272 and *m*/*z* 230 in the corresponding mass spectra of compounds (in negative ion mode) **L6** and **L7,** respectively, are ascribed to the respective M–H species. The ESI mass spectra of the compounds are provided in the Supplementary Materials.

### 3.2. Biochemistry: Evaluation of Antioxidant Properties

In biological systems, the free metal ions catalyze the oxidation of ascorbic acid and promote the formation of reactive oxygen species (ROS) such as superoxide radicals (O_2_^•−^), H_2_O_2_, and hydroxyl radicals (^•^OH) [67]. These reactive oxygen species damage the key molecules of biological significance, such as phospholipids, proteins, and DNA, causing oxidative stress, which is responsible for numerous health disorders such as cancer, cardiovascular disease, atherosclerosis, and Alzheimer’s disease [68]. Therefore, the study of compounds possessing high free radical scavenging activity is of immense research interest [67].

In the present work, we have used three different methods to study the antioxidant activity of edaravone derivatives and their Cu(II) complexes. The methods we applied are from the respective groups of hydrogen atom transfer (DPPH), electron transfer (ABTS) [69,70], and ROS (H_2_O_2_) [68].

#### 3.2.1. Hydrogen Peroxide Scavenging Activity (HPSA) Assay

**Figure 2:**
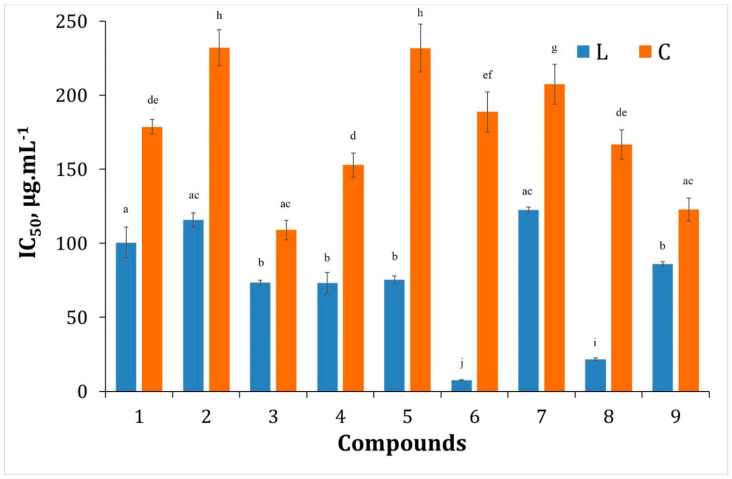
Results of the HPSA of edaravone derivatives (**L1**–**L9**) and their Cu(II) complexes (**C1**–**C9**). Values are presented as IC_50_, µg.mL^−1^. Ascorbic acid (IC_50_ = 24.84 µg.mL^−1^) was used as a standard. Different letters for the same method indicate significant difference at *p* < 0.05 levels by Duncan’s test. Duncan’s test allows us to use edaravone (**L1**) as a standard by which to compare the antioxidant activity of its derivatives (**L1**–**L9**) and Cu(II) complexes (**C1**–**C9**).

The inflammatory process accelerates the formation of ROS, especially superoxide, which will lead to the formation of H_2_O_2_ (superoxide dismutation) and which will further form hydroxyl radicals (the Haber–Weiss process) [71]. Removal of H_2_O_2_ is very important in preventing the generation of highly reactive hydroxyl radicals. Therefore, in the present work, the H_2_O_2_ scavenging property of the synthesized edaravone compounds is investigated and compared with the standard, edaravone (**L1**), and with ascorbic acid. Figure 2 depicts the bar diagram of the hydrogen peroxide scavenging activity (HPSA) analysis data (The IC_50_ values of the compounds are listed in Table S1 in the Supplementary Materials). The IC_50_ values of the synthesized compounds ranged from 7.61 to 232.10 µg.mL^−1^. Compared with ascorbic acid, edaravone derivatives **L6** and **L8** are characterized by higher antioxidant activity. The statistical evaluation of the antioxidant activity of the edaravone derivatives and their complexes was conducted using Duncan’s test. In this case, we used edaravone (**L1**) as a standard by which to distinguish the individual groups. Duncan’s test enables the evaluation of differences in antioxidant activity, while also assessing how the activity varies based on the structure of the compounds. Only the compounds, **L6** (7.61 µg.mL^−1^) and **L8** (21.61 µg.mL^−1^), which contain semicarbazone and thiocyanate substituents, respectively, were found to have lower IC_50_ values than the standard, edaravone (**L1**), indicating their prominent antioxidant activity. Interestingly, all of the organic edaravone derivatives exhibited higher antioxidant activity than their respective Cu(II) complexes, varying from 1.4 to a striking 24 times higher activity.

#### 3.2.2. DPPH Free Radical Scavenging Activity Assay

The DPPH (2,2-diphenyl-1-picrylhydrazyl) radical scavenging activity is a widely used method for the investigation of the free radical scavenging ability of newly developed compounds. Change in the color of the DPPH radical from violet to yellow upon reduction is monitored in this test [72].

**Figure 3:**
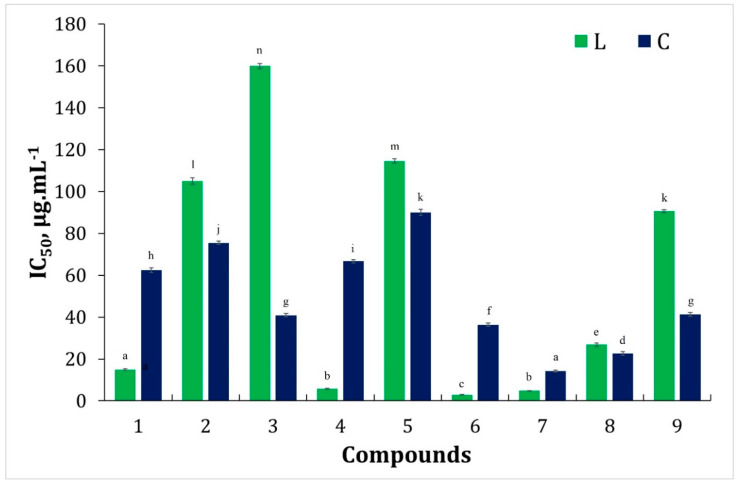
Results of DPPH assay of edaravone derivatives (**L1**–**L9**) and their Cu(II) complexes (**C1**–**C9**). Values are presented as IC_50_, µg.mL^−1^. Edaravone (**L1**) was used as a standard. Different letters for the same method indicate significant difference at *p* < 0.05 levels by Duncan’s test.

The results for radical scavenging activity of the synthesized compounds are presented in Figure 3 (The IC_50_ values of the compounds are listed in Table S1 in the Supplementary Materials). The edaravone derivatives **L4**, **L6**, and **L7** are characterized by significantly high radical scavenging activity, with IC_50_ values ranging from 2.94 to 5.84 µg.mL^−1^. Their higher activity may be due to the fact that they more easily donate a proton for subsequent reaction with DPPH. A medium level activity is shown by the compounds **L1** and **L8,** with IC_50_ values between 13 and 26 µg.mL^−1^. While the compounds **L2**, **L3**, **L5** and **L9** showed low antioxidant activity. Among the corresponding complexes, **C7** (IC_50_ = 14.15 µg.mL^−1^) and **C8** (IC_50_ = 22.55 µg.mL^−1^) exhibited good antioxidant activity. Interestingly, the Cu(II) complexes **C2**, **C3**, **C5** and **C9** showed higher antioxidant activity compared with their corresponding ligands, indicating the involvement of a copper center in the antioxidant activity [38,39,40,41].

#### 3.2.3. ABTS Assay

**Figure 4:**
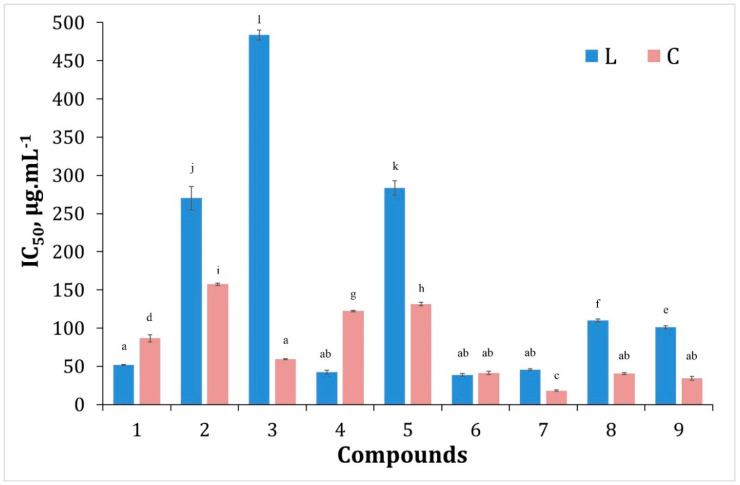
Results of ABTS assay of edaravone derivatives (**L1**–**L9**) and their Cu(II) complexes (**C1**–**C9**). Values are presented as IC_50_, µg.mL^−1^. Edaravone (**L1**) was used as a standard. Different letters for the same method indicate significant difference at *p* < 0.05 levels by Duncan’s test.

The results determined by ABTS (2,2′-azino-bis(3-ethylbenzothiazoline-6-sulfonic acid) analysis are presented in Figure 4 (The IC_50_ values of the compounds are listed in Table S1 in the Supplementary Materials). This is a widely used method for the determination of the total antioxidant capacity of the compounds. Decolorization of the blue/green ABTS^•+^ radical by the act of antioxidants is monitored in this assay [73]. In the current investigation, the organic compounds **L1**, **L4**, **L6**, and **L7** exhibited high radical scavenging activity with IC_50_ values ranging from 38.70 to 51.88 µg.mL^−1^. The copper(II) complexes **C3**, **C6**, **C7**, **C8**, and **C9** showed significantly higher radical activity than the corresponding ligands, with the complex **C7** (IC_50_ = 18.23 µg.mL^−1^) exhibiting the highest radical scavenging activity among all of the tested compounds.

### 3.3. Computational Studies

#### 3.3.1. Electronic Properties

**Figure 5:**
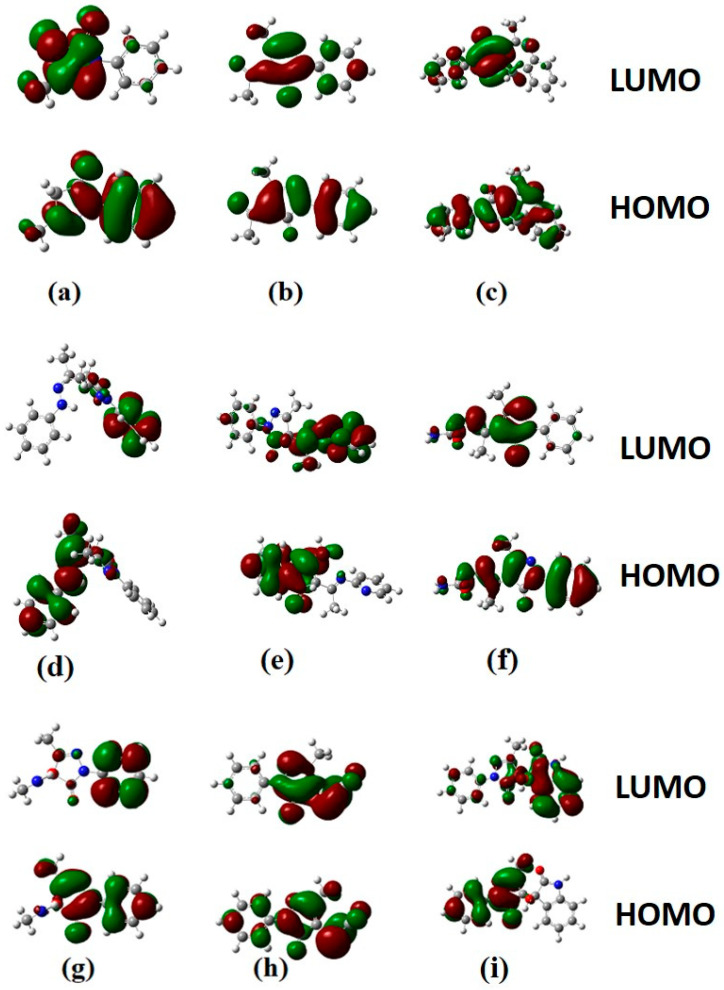
HOMO–LUMO plots of DFT optimized structures of (**a**) **L1**, (**b**) **L2**, (**c**) **L3**, (**d**) **L4**, (**e**) **L5**, (**f**) **L6**, (**g**) **L7**, (**h**) **L8**, and (**i**) **L9**.

From the three assays used for the evaluation of antioxidant activities of the compounds, it is observed that the organic compounds generally exhibited better antioxidant activities as compared with the corresponding copper(II) complexes. Hence, for further computational studies, only the organic compounds were considered. The chemical characteristics of the organic ligands were calculated theoretically to anticipate a comparison of antioxidant activity exhibited by them. The results are presented in Table 1. The frontier molecular orbital (FMO) energies are crucial in establishing the chemical stability and reactivity of the molecules [74]. The HOMO–LUMO plots of the organic compounds **L1**–**L9** are given in Figure 5. The charge transfer in the systems is determined by the distribution of electron densities in the HOMO–LUMO plots of all of the organic molecules. A large energy gap of about 5.2351 eV is computed for the compound **L2**, while the other compounds are found to be in the range of 2.7697–5.2318 eV, suggesting higher reactivity for the latter group than the former, which is a good indicator of reactivity. The LUMO energies obey the downward order, as follows: −0.7129 (**L6**) > −0.7377 (**L7**) > −0.9448 (**L2**) > 1.0182 (**L3**) > −1.0776 (**L8**) > −1.0781 (**L1**) > −1.1684 (**L9**) > −1.1924 (**L5**), whereas the HOMO energies follow the range −5.3959 (**L4**) to −6.3094 (**L8**), reflecting the decreasing and the increasing of the electron acceptor and donor roles, respectively.

**Figure 6:**
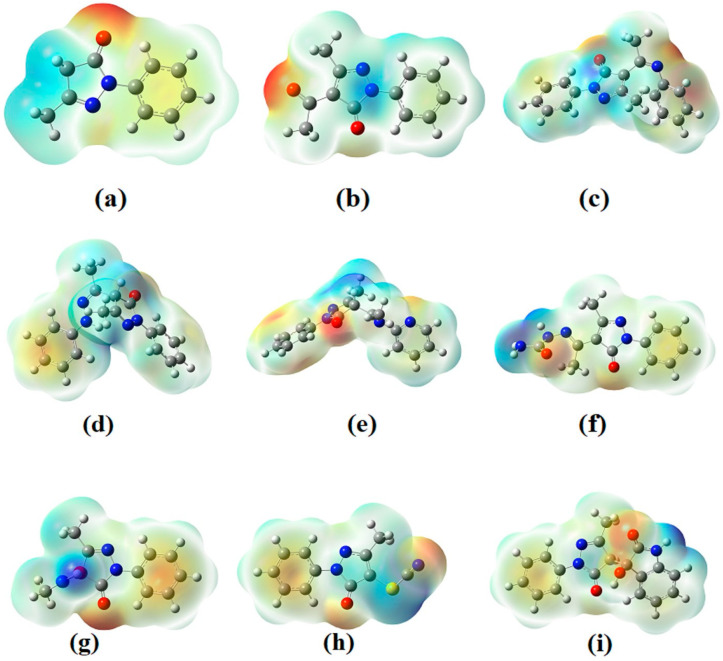
The molecular electrostatic potential (MEP) plots of the organic compounds (color code: carbon – grey, nitrogen – blue, oxygen – red, hydrogen – white, sulfur – yellow) (**a**) **L1**, (**b**) **L2**, (**c**) **L3**, (**d**) **L4**, (**e**) **L5**, (**f**) **L6**, (**g**) **L7**, (**h**) **L8**, and (**i**) **L9**.

The electrophilicity varies inversely to the energy gap and the chemical hardness, but it increases with the augmentation of the square of the chemical potential. The high electrophilicity value is related to the relatively smaller energy gap. From Table 2, the possible reactivity order is established as follows: 2.0722 (**L6**) < 2.0794 (**L7**) < 2.4240 (**L2**) < 2.4472 (**L3**) < 2.5124 (**L1**) < 2.6412 (**L9**) < 2.6447 (**L5**) < 2.6075 (**L8**) < 2.8656 (**L4**). The molecular electrostatic potential (MEP) plots of the organic compounds **L1**–**L9** are presented in Figure 6. The plots show the isosurfaces of nucleophilic and electrophilic attack sites. The red and yellow zones represent the electronegative and blue regions are electropositive zones. From the MEP surfaces, it is very clear that most of the molecules possess reactive sites.

#### 3.3.2. Molecular Dynamics and Docking

**Figure 7:**
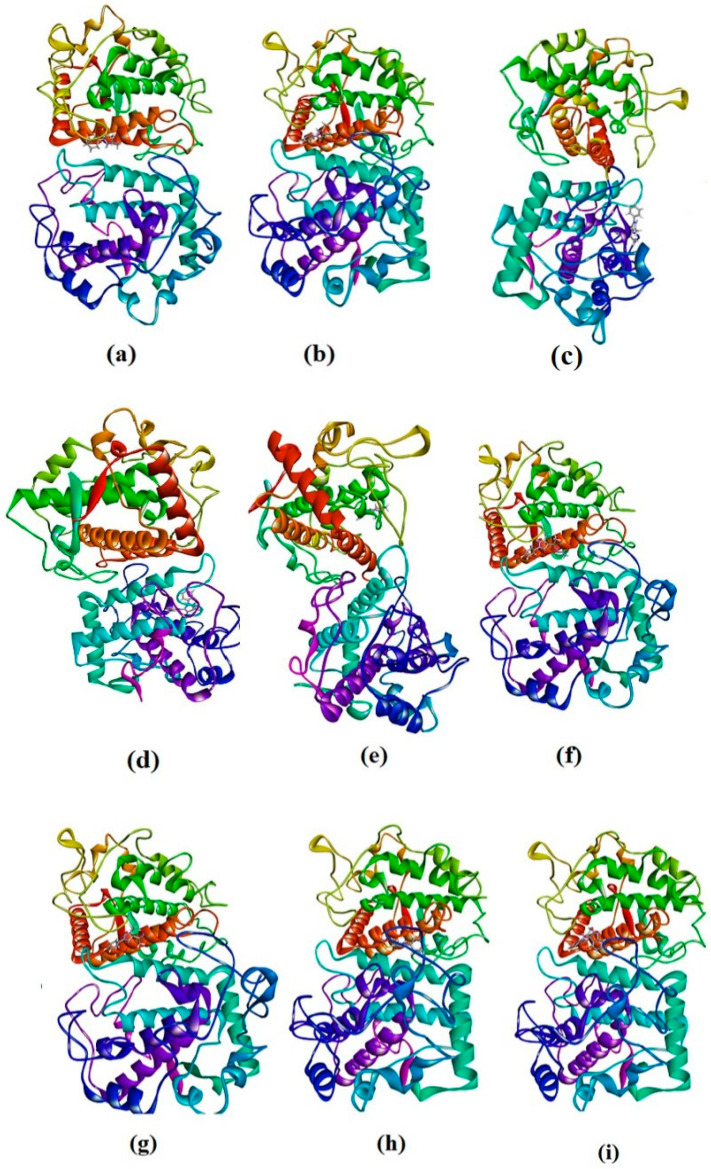
Interaction plots of **3NM8** with (**a**) **L1**, (**b**) **L2**, (**c**) **L3**, (**d**) **L4**, (**e**) **L5**, (**f**) **L6**, (**g**) **L7**, (**h**) **L8**, and (**i**) **L9**.

To study the biological activity of compounds and activity functions, the most widely used techniques in drug discovery are docking and MD simulations [75]. These predict the binding modes and the stability of the complex by its corresponding interactions and binding affinity [76]. As there is a considerable interest in evaluating the tyrosinase inhibitory activity of the newly developed antioxidant compounds, we investigated the docking and binding affinities of our compounds with tyrosinase from *Bacillus megaterium* (PDB ID: **3NM8**) [77,78]. The binding energies of each compound are tabulated in Table 2. The interaction plots for organic ligands **L1**–**L9** are represented in Figure 7. The binding energies for the organic ligands (**L1**–**L9**) are found to be in the range −6.9 (i.e., **L5**) to −11.5 kcal/mol (i.e., **L9**). As, in the experimental results (*vide supra*), the organic molecule **L6** (binding energy −9.4 kcal/mol) has exhibited comparatively better antioxidant activity among the tested compounds, it was selected for further molecular dynamics (MD) simulation studies.

A complex with a selected ligand, **L6,** was subjected to molecular dynamics simulation analysis. The MD simulation was performed (150 ns) to estimate the stability of the **3NM8–L6** complex, investigation of other parameters, such as root mean square deviation (RMSD), root mean square fluctuation (RMSF), radius of gyration (Rg), hydrogen bonds, and solvent accessible surface area (SASA). Additionally, molecular mechanics Poisson–Boltzmann surface area (MMPBSA) analysis was performed.

**Figure 8:**
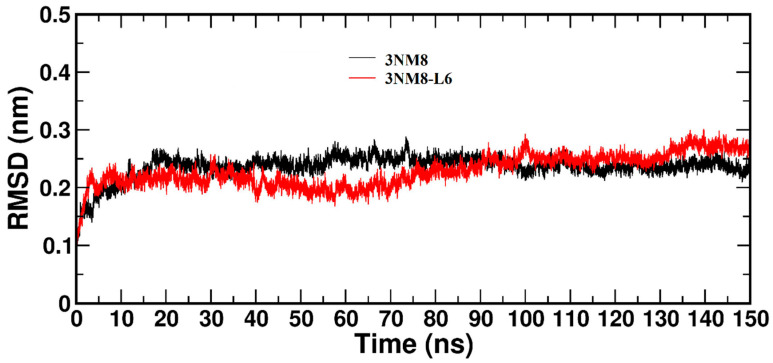
Root mean square deviation of the backbone atoms of **3NM8** and **3NM8–L6** complex.

**Figure 9:**
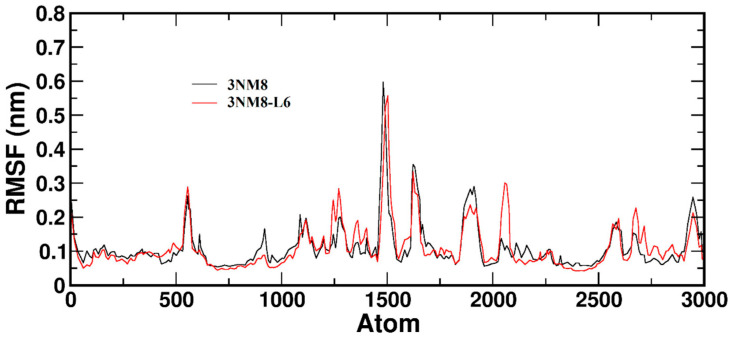
Root mean square fluctuation of the c-alpha atoms of **3NM8** and **3NM8–L6**.

**Figure 10:**
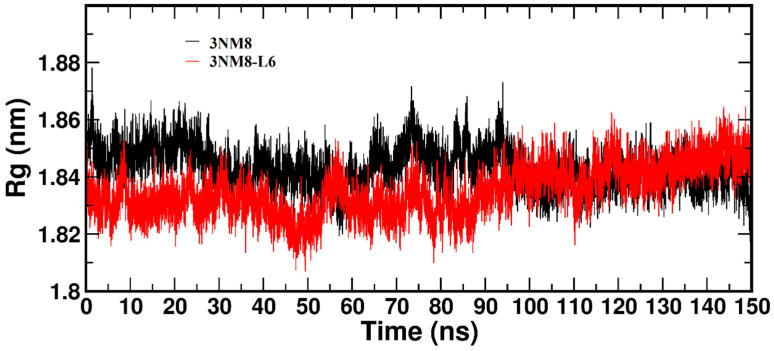
Rg of the backbone atoms of **3NM8** and **3NM8–L6**.

**Figure 11:**
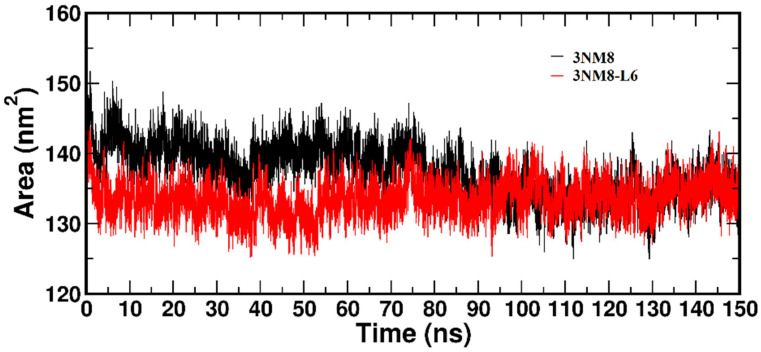
SASA of the backbone atoms of **3NM8** and **3NM8–L6**.

**Figure 12:**
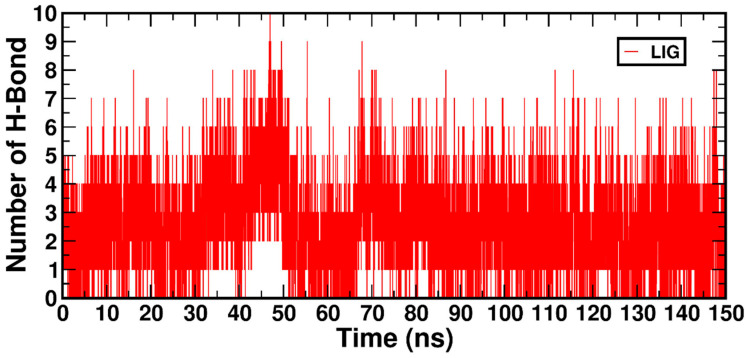
H-bonds of complex **3NM8–L6**.

**Figure 13:**
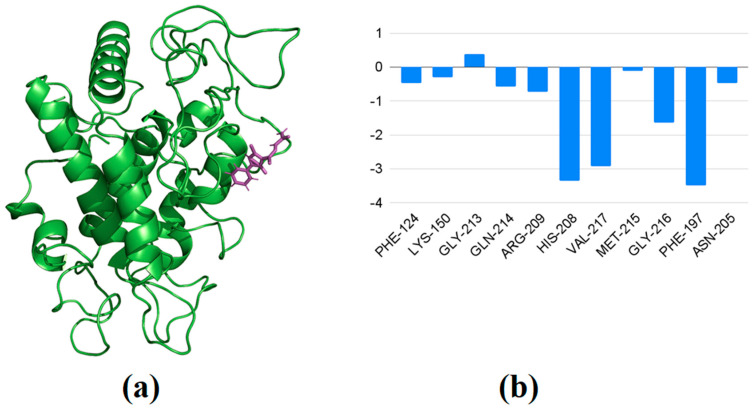
(**a**) Last frame of MD trajectory (150 ns). (**b**) Active site MMPBSA values of **3NM8–L6.**

RMSD is a parameter used to estimate conformation differences, giving higher RMSD values for higher deviation. The average RMSD values (0 to 150 ns) obtained for the **3NM8** and **3NM8–L6** complex proteins are 0.21 nm and 0.22 (Figure 8), respectively. These values suggest the relative stability of the **3NM8–L6** complex during the entire simulation [79]. RMSF determines which amino acids of the protein **3NM8** produce large vibrations, destabilizing **3NM8** in the presence and absence of the **L6.** The RMSF (0 to 150 ns) for **3NM8** and **3NM8–L6** is depicted in Figure 9 [80]. The Rg value determines the compactness of **3NM8** and the folding and unfolding with **L6**. The average Rg (0 to 150 ns) for **3NM8** and **3NM8–L6** were found to be 1.05 nm [81]. The Rg of **3NM8** and **3NM8–L6** are depicted in Figure 10. SASA influences the flexibility and compactness of the protein **3NM8.** To understand the modulation of inhibitors, the changes in the SASA values upon binding with compound **L6** was analyzed. The SASA values ranging from 0 to 150 are shown in Figure 11. The changes are negligible in the present case, indicating that the binding of **L6** does not affect the surface area of the receptor protein significantly [82]. The **3NM8–L6** complex is stabilized by H-bond formation, which is confirmed by docking and MD analysis, as depicted in Figure 12. MMPBSA gives information regarding the energy required for the **L6** to bind to **3NM8** and, in the present study, the van der Waals, electrostatic, polar solvation and binding energies are found to be −107.251 ± 43.640, −11.999 ± 9.586, 58.483 ± 27.579 and −71.450 ± 38.951 kJ/mol, respectively (see Figure 13).

## 4. Conclusions

In conclusion, this work reports the synthesis and characterization of functional derivatives of edaravone and their copper(II) complexes, along with the assessment of their antioxidant and melanogenic inhibiting activity. This study provides an insight into the correlation of structural features of the new edaravone derivatives and their activities. Molecular structures of the synthesized organic compounds and corresponding copper(II) complexes were determined based on the spectral and analytical characterization methods, including IR, NMR, UV–vis, mass spectrometry and elemental analysis. The antioxidant activity of all of the compounds was evaluated by HPSA, DPPH and ABTS assays. In the HPSA assay, the edaravone-derivative-containing semicarbazone group, i.e., **L6,** exhibited the highest activity, with IC_50_ = 7.61 µg.mL^−1^. Remarkably, the organic compounds were found to be better antioxidants in this assay as compared with the corresponding copper(II) complexes. In the DPPH assay, the organic derivatives **L4**, **L6**, and **L7** exhibited highest radical scavenging activity (IC_50_ = 2.94–5.84 µg.mL^−1^), which is attributed to their proton-donating ability to DPPH. In the ABTS assay, the best of organic compounds **L1**, **L4**, **L6**, and **L7** exhibited IC_50_ values in the range of 38.70–51.88 µg.mL^−1^, while the corresponding copper(II) complexes showed considerably higher activity, the complex **C7**, which contains an edaravone–oxime ligand exhibited the finest activity (i.e., IC_50_ = 18.23 µg.mL^−1^). Owing to their higher antioxidant activity, all of the organic ligands were subjected to DFT analysis to determine the frontier orbital energy levels as well as important chemical properties, such as band gap, hardness, chemical potential and electrophilicity index. A clear influence of substituents on these properties was observed. The reactive sites on the molecules were identified using MEP plots. Owing to their remarkable antioxidant activities, the potential anti-melanogenic properties of all of the organic compounds was investigated by docking the DFT optimized structures with the protein tyrosinase. All of the compounds exhibited favorable binding energies, indicating their potential use as anti-melanogenic agents. Further, the derivative, **L6**, which exhibited the best antioxidant activity, was further subjected to molecular dynamics simulation analysis. The MD simulation was performed (150 ns) to estimate the stability of a protein–ligand complex (i.e., **3NM8–L6**), and other parameters, such as RMSD, RMSF, Rg, hydrogen-bond interactions, SASA and MMPBSA were analyzed. Overall, this new series of compounds exhibited strong free radical scavenging activity and shown to possibly prevent melanogenesis by strongly binding with tyrosinase protein, indicating their potential application in the production of improved antioxidants and cosmeceuticals.

## Figures and Tables

**Table 1:** The frontier orbital energy levels and chemical properties of the compounds derived from the DFT studies.

Compound	E_HOMO_ (eV)	E_LUMO_ (eV)	E_LUMO_–E_HOMO_ Gap (eV)	Hardness	Chemical Potential	Electrophilicity Index
**L1**	−5.8392	−1.0781	4.7611	2.3806	−3.4587	2.5124
**L2**	−6.1799	−0.9448	5.2351	2.6176	−3.5624	2.4240
**L3**	−5.8819	−1.0182	4.8637	2.4319	−3.4501	2.4472
**L4**	−5.3959	−1.3848	4.0111	2.0056	−3.3904	2.8656
**L5**	−5.7557	−1.1924	4.5633	2.2817	−3.4741	2.6447
**L6**	−5.7461	−0.7129	5.0332	2.5166	−3.2295	2.0722
**L7**	−5.6623	−0.7377	4.9246	2.4623	−3.2000	2.0794
**L8**	−6.3094	−1.0776	5.2318	2.6159	−3.6935	2.6075
**L9**	−5.9097	−1.1684	4.7413	2.3707	−3.5391	2.6416

**Table 2:** Binding energies of synthesized compounds with the protein tyrosinase from *Bacillus megaterium* (PDB ID: **3NM8**).

Compounds	Binding Energy (kcal/mol)
**L1**	−7.8
**L2**	−9.2
**L3**	−7.6
**L4**	−7.3
**L5**	−6.9
**L6**	−9.4
**L7**	−8.9
**L8**	−8.0
**L9**	−11.5

## Data Availability

The original contributions presented in the study are included in the article/Supplementary Materials, further inquiries can be directed to the corresponding author/s.

## Supplementary Materials

The following supporting information can be downloaded at: https://www.mdpi.com/article/10.3390/antiox13091148/s1, IR, UV-vis, NMR, and Mass spectroscopic data of the reported compounds and their antioxidant activity results (HPSA, DPPH, and ABTS assays).
