# Supplementary material for: Synthesis, Characterization and Assessment of Antioxidant and Melanogenic Inhibitory Properties of Edaravone Derivatives
Source: Antioxidants (Basel). 2024 Sep 23;13(9):1148. doi: 10.3390/antiox13091148 (PMC11429142; doi:10.3390/antiox13091148)
Supplement: Supplementary file 1 [file antioxidants-13-01148-s001.zip › antioxidants-3212818-supplementary.pdf]

Supplementary data

Synthesis, Characterization and Assessment of Antioxidant and Melanogenic Inhibitory Properties of Edaravone Derivatives

**R. Divya Mohan [1], S. A. Anaswara [1], Naveen V. Kulkarni [1,*], Dimitar G. Bojilov [2], Stanimir P. Manolov [2,*],
Iliyan I. Ivanov [2], Jamelah S. Al-Otaibi [3] and Y. Sheena Mary [4]**

[1] Department of Chemistry, Amrita Vishwa Vidyapeetham, Amritapuri 690525, India; divyamohanr@am.amrita.edu (R.D.M.); anaswaraplr@gmail.com (S.A.A.)
[2] Department of Organic Chemistry, University of Plovdiv, 24 Tzar Assen str., 4000 Plovdiv, Bulgaria; bozhilov@uni-plovdiv.net (D.G.B.); iiiliyan@abv.bg (I.I.I.)
[3] Department of Chemistry, College of Science, Princess Nourah bint Abdulrahman University,
P.O. Box 84428, Riyadh 11671, Saudi Arabia; dr.jamelah@gmail.com
[4] Department of Physics, FMNC, University of Kerala, Kollam, 691001 India; sheena@fatimacollege.net
* Correspondence: authors' email: naveenvkulkarni@am.amrita.edu (N.V.K.); manolov@uni-plovdiv.bg (S.P.M.)

IR Spectra of organic compounds and copper complexes

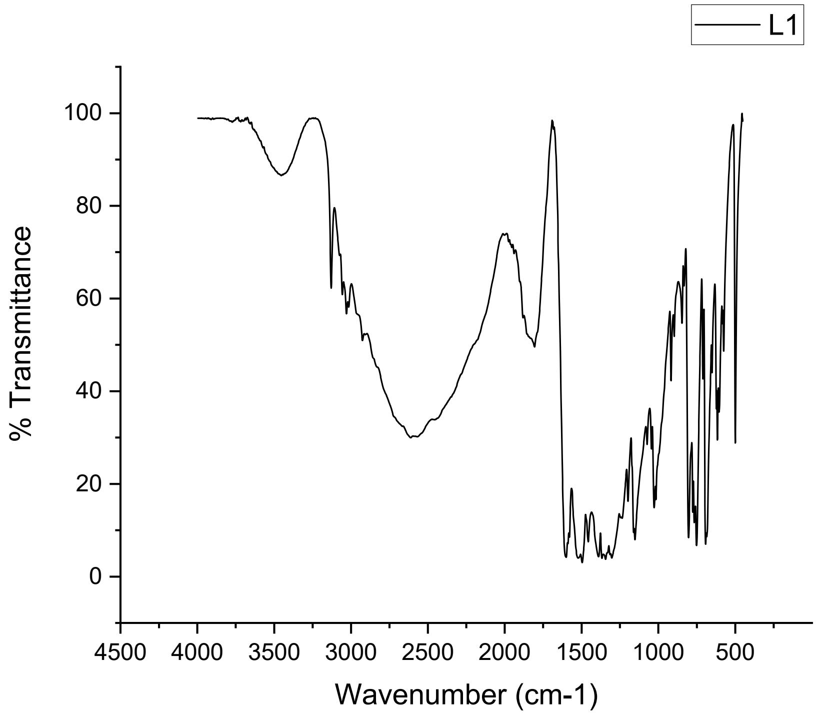

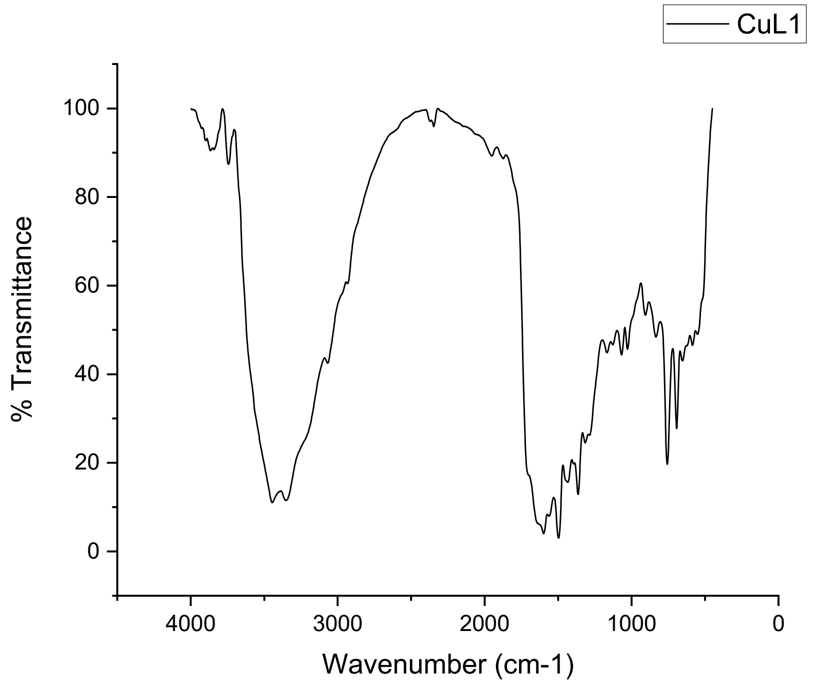

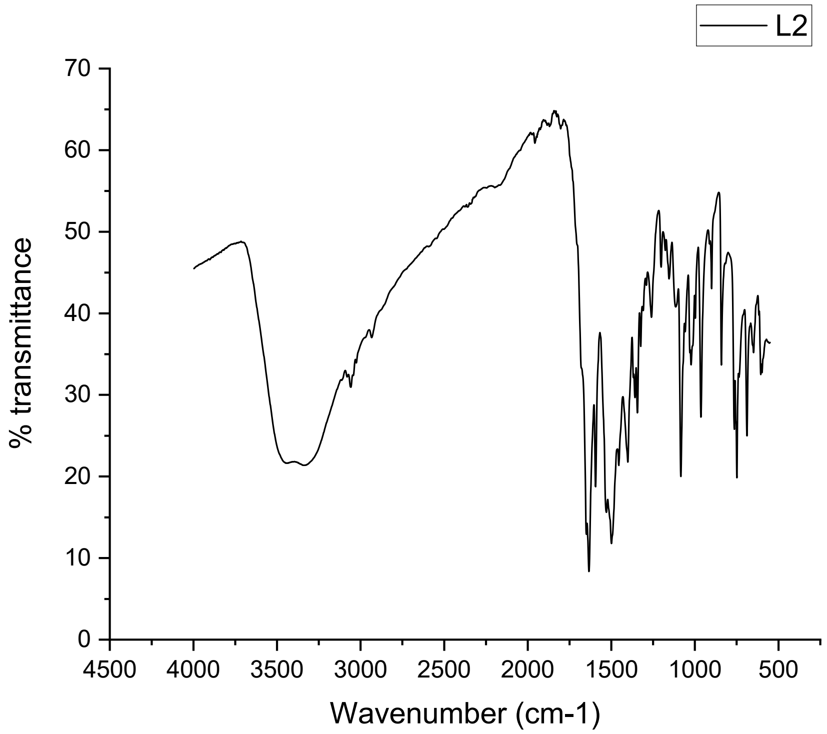

L2
% transmittance
Wavenumber (cm-1)
0
10
20
30
40
50
60
70
4500
4000
3500
3000
2500
2000
1500
1000
500

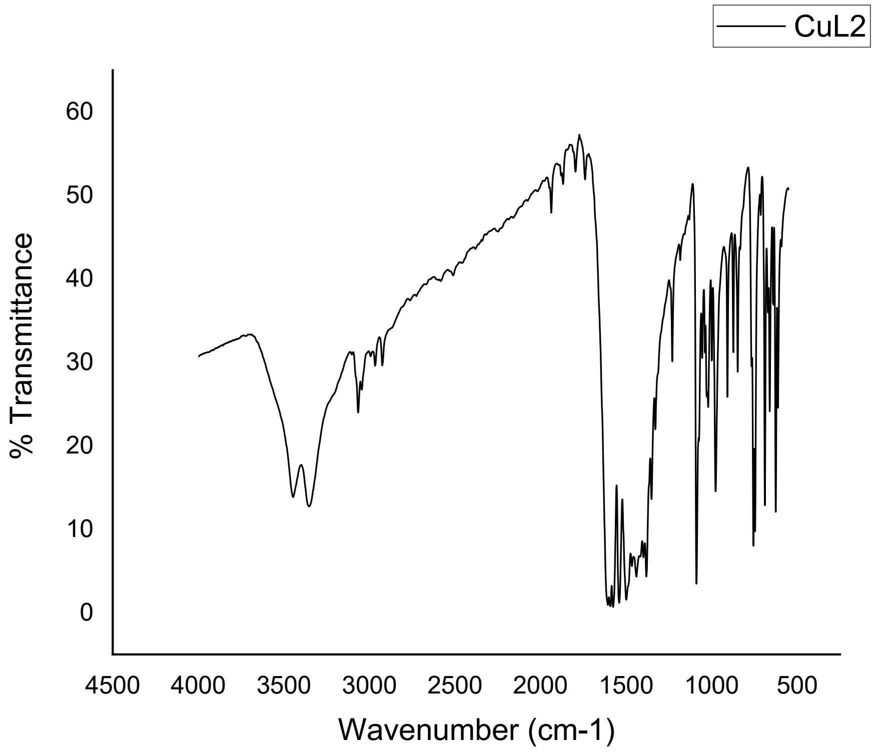

CuL2
% Transmittance
Wavenumber (cm-1)
0
10
20
30
40
50
60
4500
4000
3500
3000
2500
2000
1500
1000
500

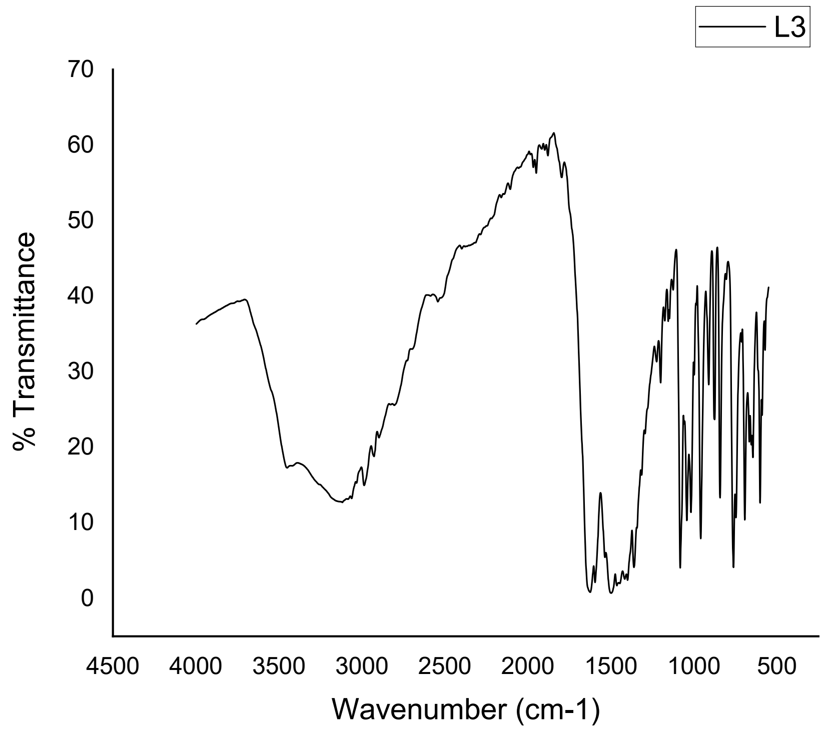
L3
70
60
50
40
30
20
10
0
% Transmittance
4500
4000
3500
3000
2500
2000
1500
1000
500
Wavenumber (cm-1)

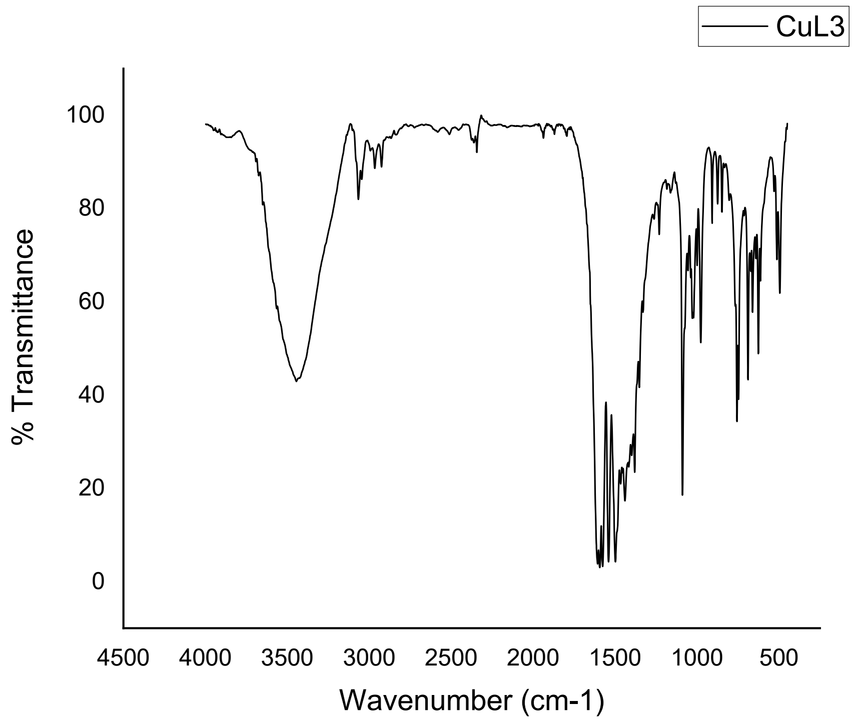
CuL3
100
80
60
40
20
0
% Transmittance
4500
4000
3500
3000
2500
2000
1500
1000
500
Wavenumber (cm-1)

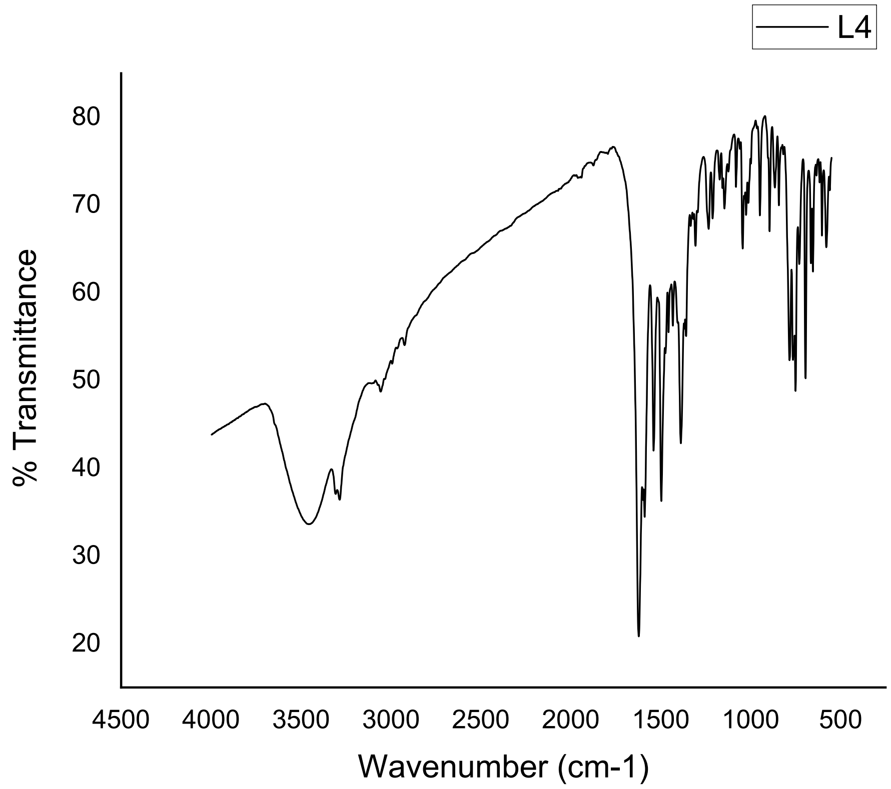
L4
80
70
60
50
40
30
20
% Transmittance
4500
4000
3500
3000
2500
2000
1500
1000
500
Wavenumber (cm-1)

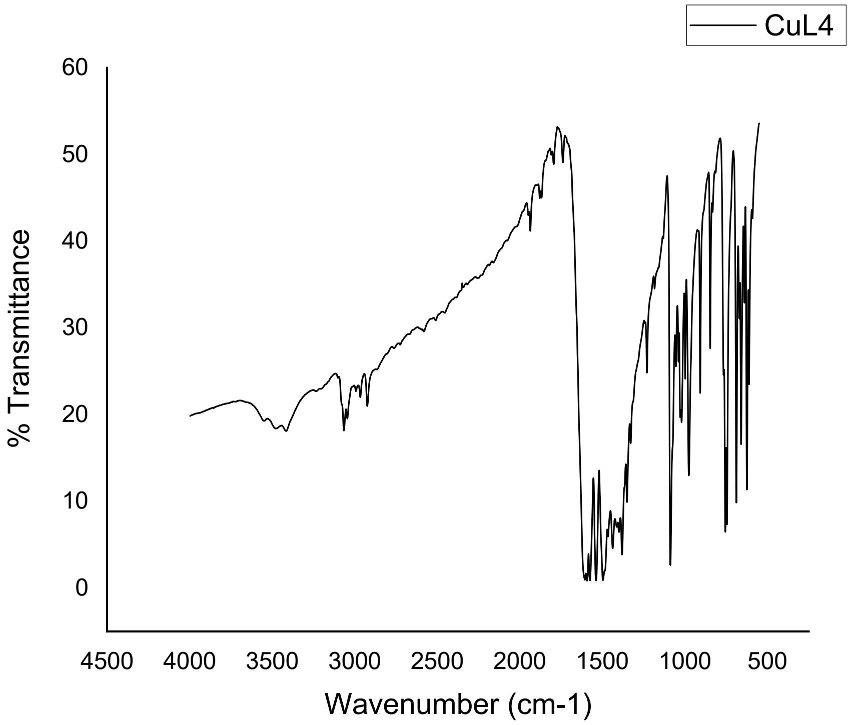
CuL4
60
50
40
30
20
10
0
% Transmittance
4500
4000
3500
3000
2500
2000
1500
1000
500
Wavenumber (cm-1)

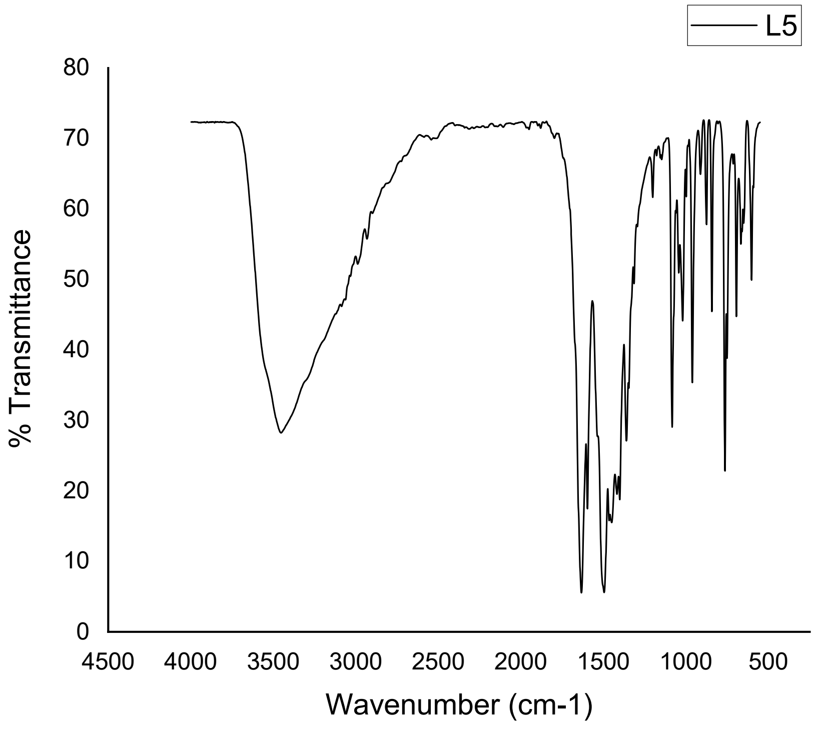
L5
% Transmittance
0
10
20
30
40
50
60
70
80
4500
4000
3500
3000
2500
2000
1500
1000
500
Wavenumber (cm-1)

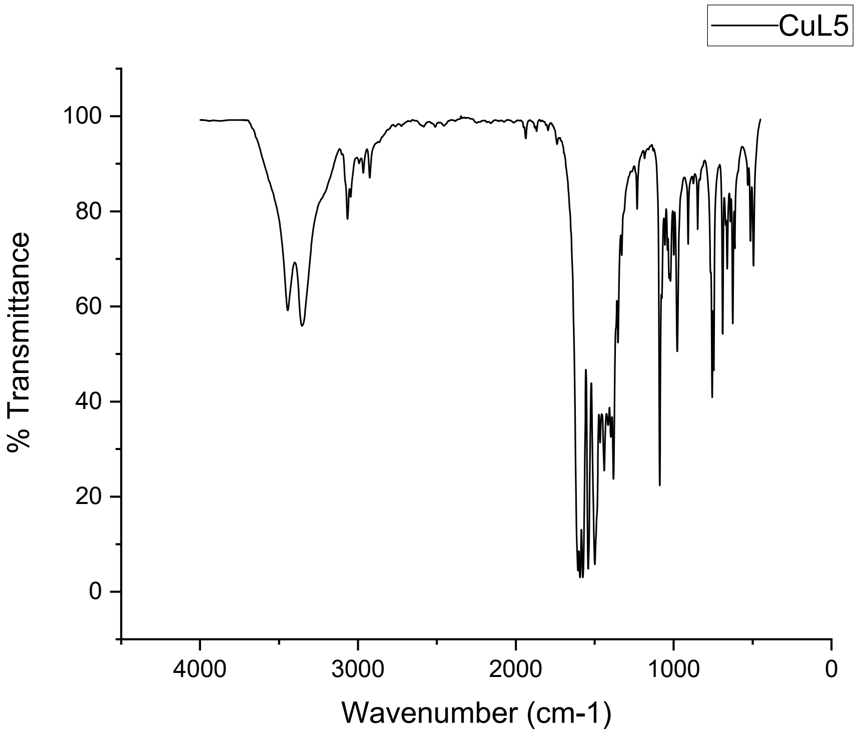
CuL5
% Transmittance
0
20
40
60
80
100
4000
3000
2000
1000
0
Wavenumber (cm-1)

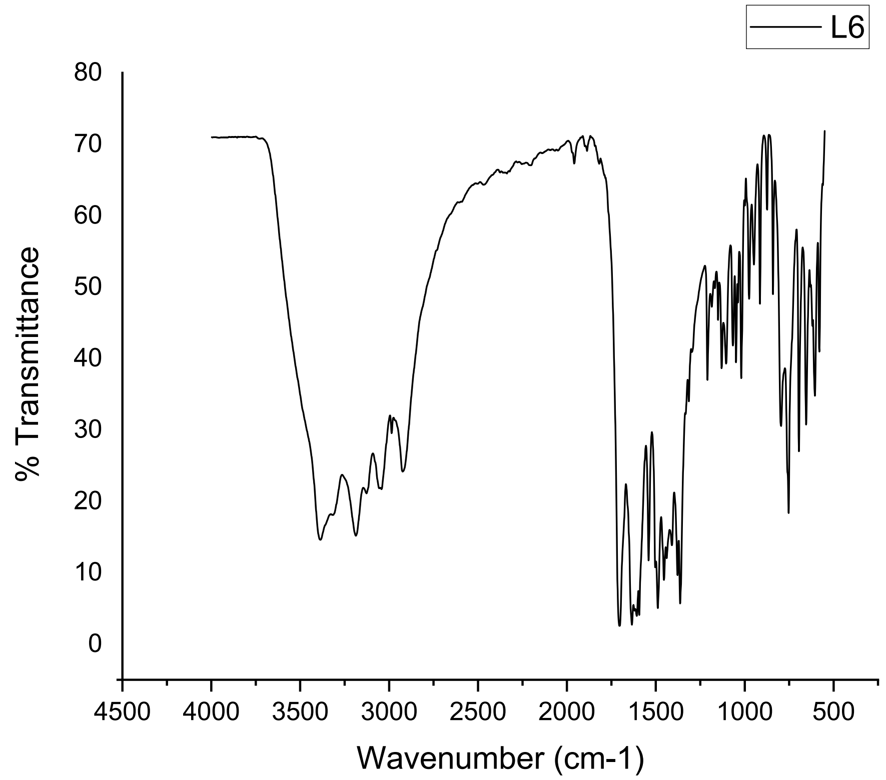
L6
% Transmittance
Wavenumber (cm-1)
80
70
60
50
40
30
20
10
0
4500
4000
3500
3000
2500
2000
1500
1000
500

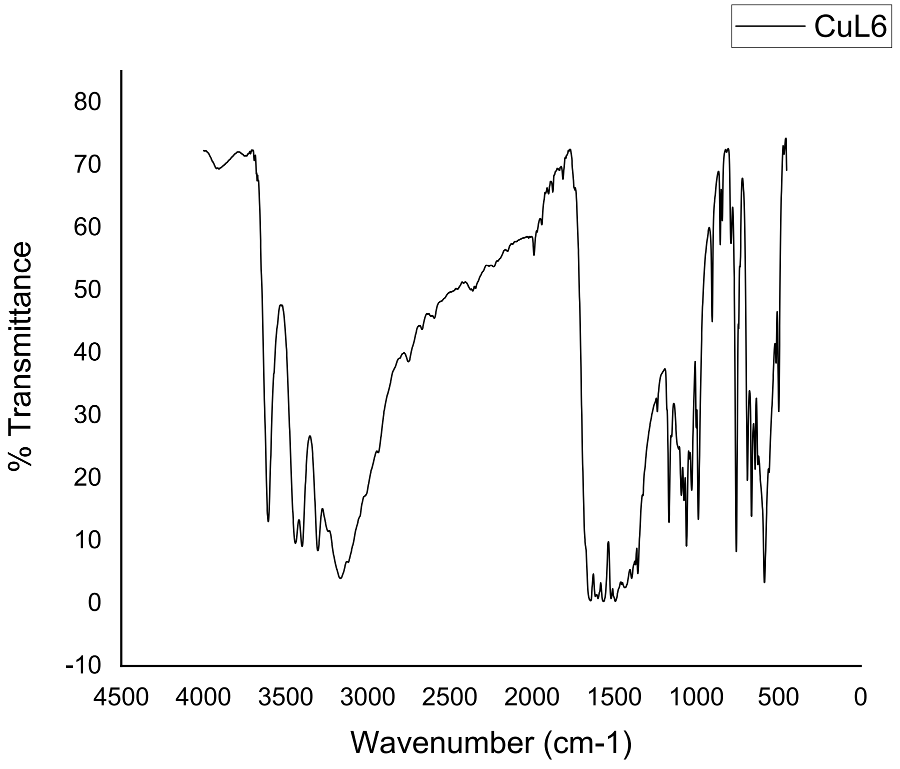
CuL6
% Transmittance
Wavenumber (cm-1)
80
70
60
50
40
30
20
10
0
-10
4500
4000
3500
3000
2500
2000
1500
1000
500
0

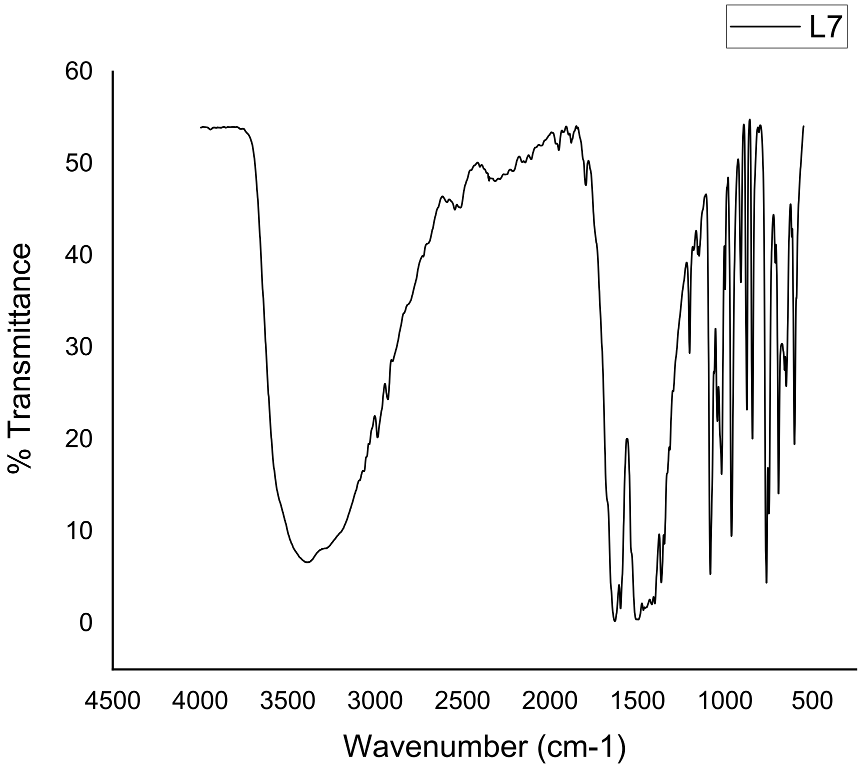
L7
% Transmittance
60
50
40
30
20
10
0
4500 4000 3500 3000 2500 2000 1500 1000 500
Wavenumber (cm-1)

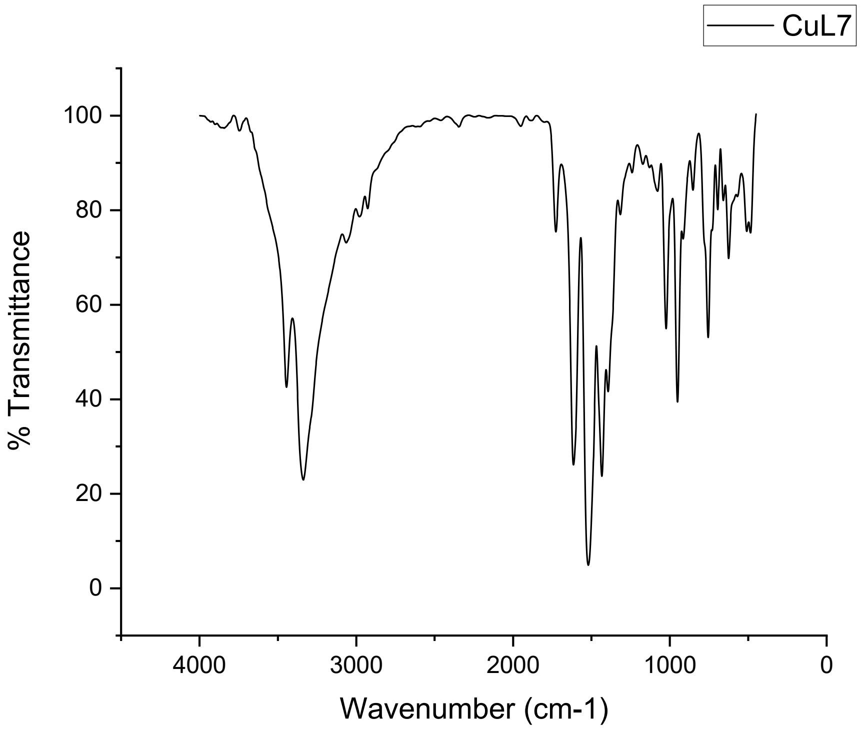
CuL7
% Transmittance
100
80
60
40
20
0
4000 3000 2000 1000 0
Wavenumber (cm-1)

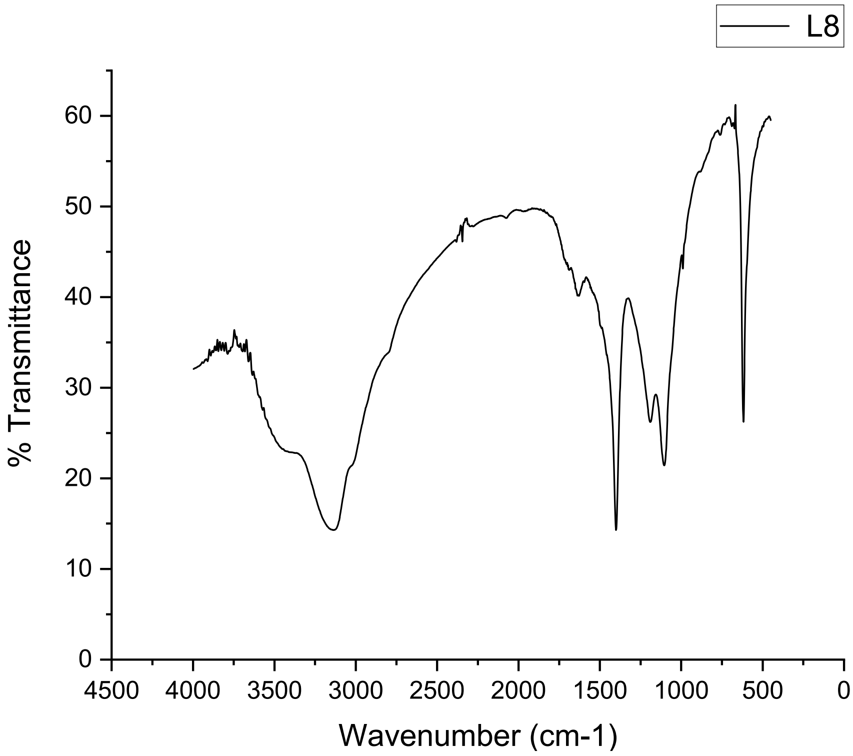
L8
% Transmittance
0
10
20
30
40
50
60
4500
4000
3500
3000
2500
2000
1500
1000
500
0
Wavenumber (cm-1)

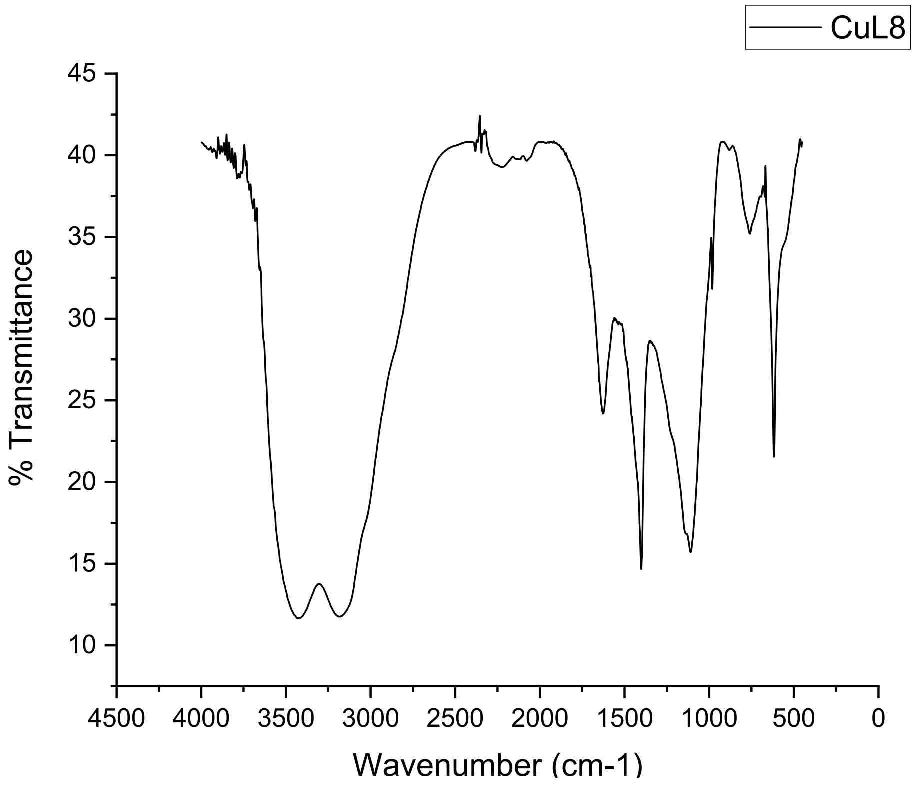
CuL8
% Transmittance
10
15
20
25
30
35
40
45
4500
4000
3500
3000
2500
2000
1500
1000
500
0
Wavenumber (cm-1)

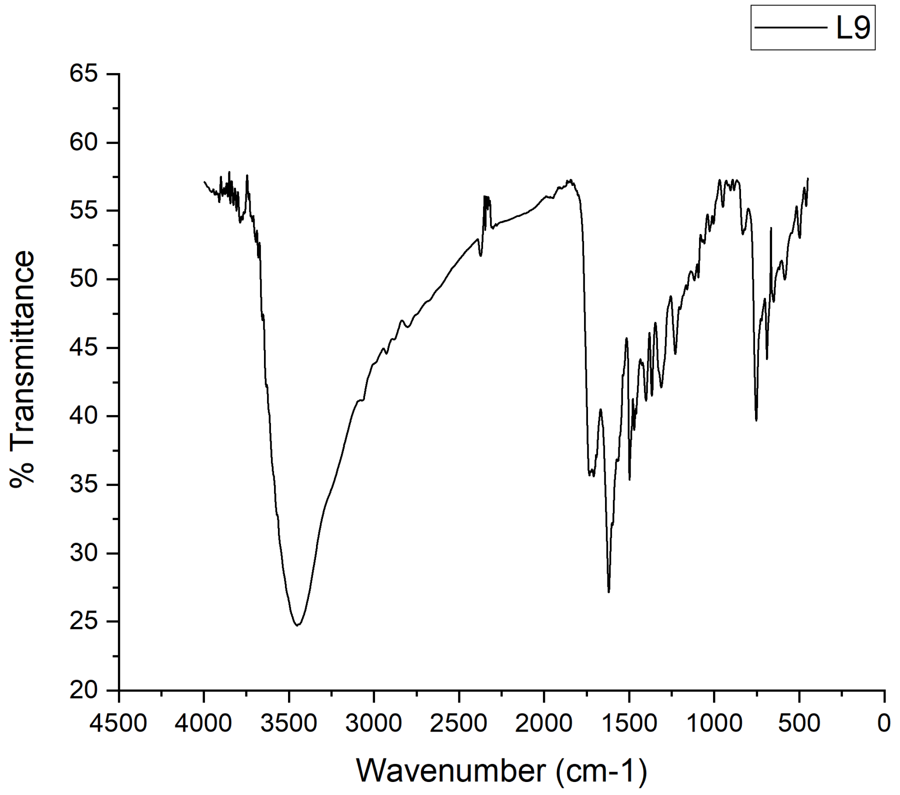
L9
% Transmittance
65
60
55
50
45
40
35
30
25
20
4500
4000
3500
3000
2500
2000
1500
1000
500
0
Wavenumber (cm-1)

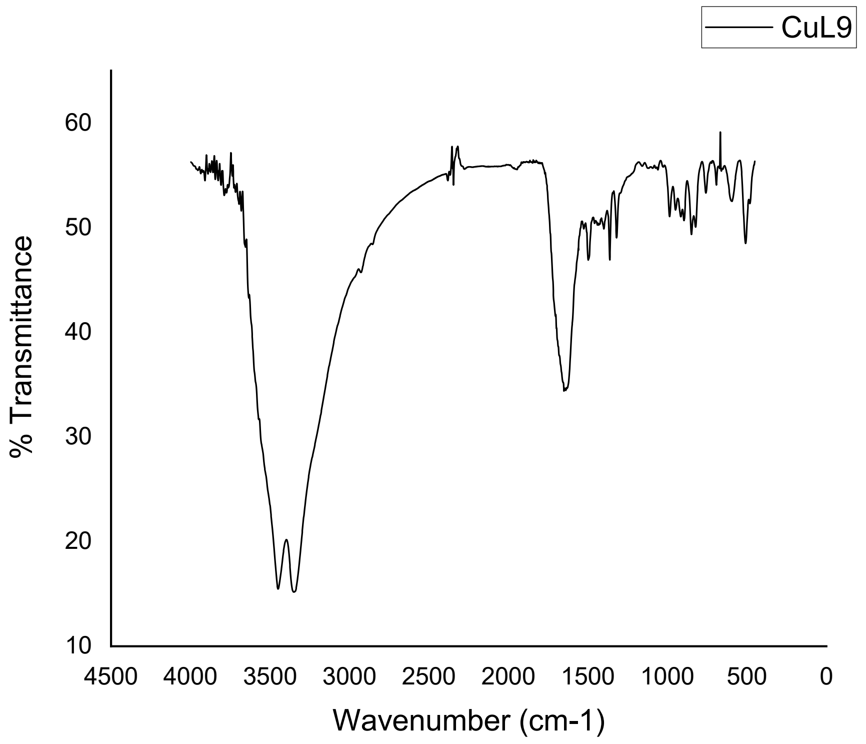
CuL9
% Transmittance
60
50
40
30
20
10
4500
4000
3500
3000
2500
2000
1500
1000
500
0
Wavenumber (cm-1)

UV-vis Spectra of organic compounds and copper complexes

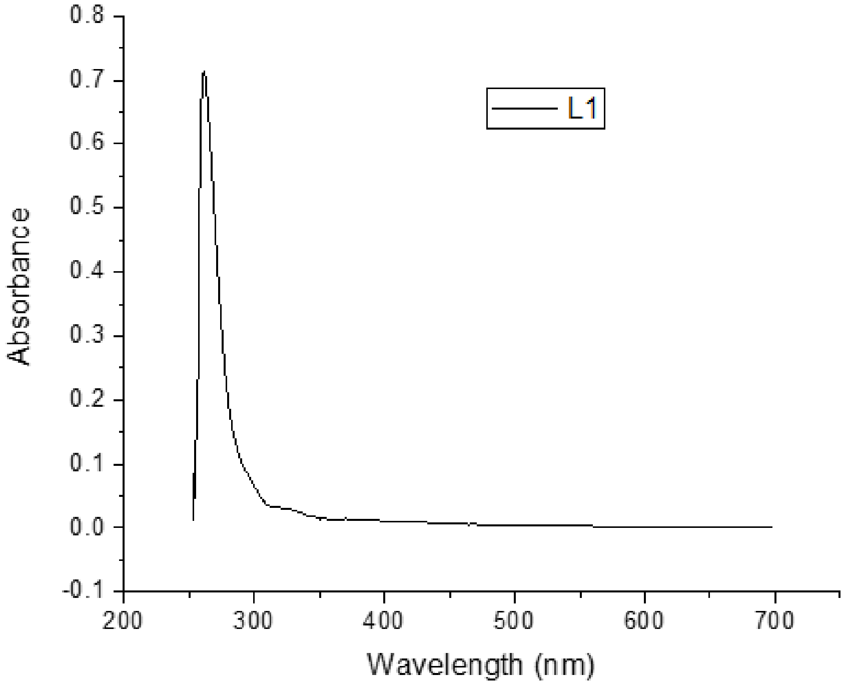

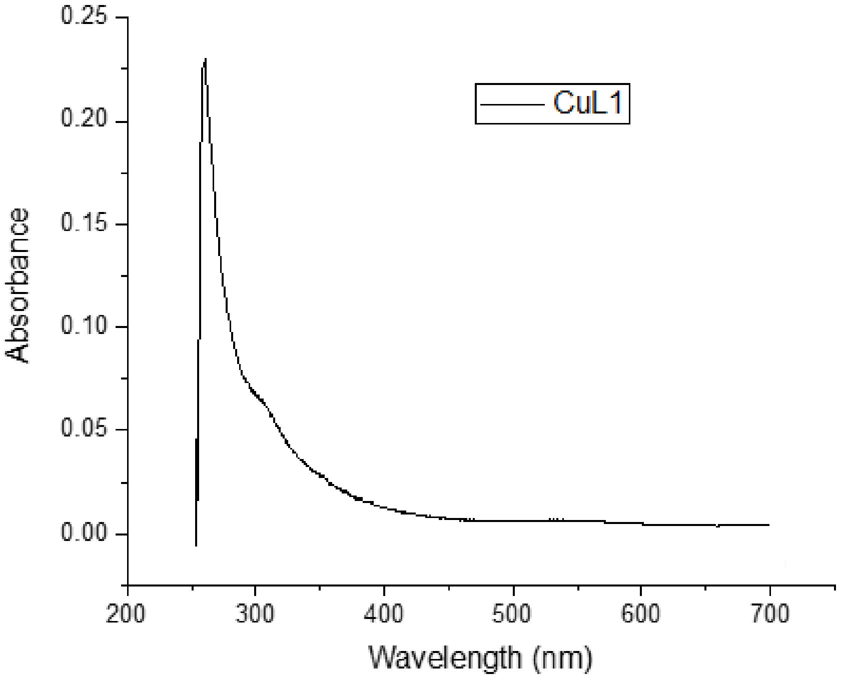

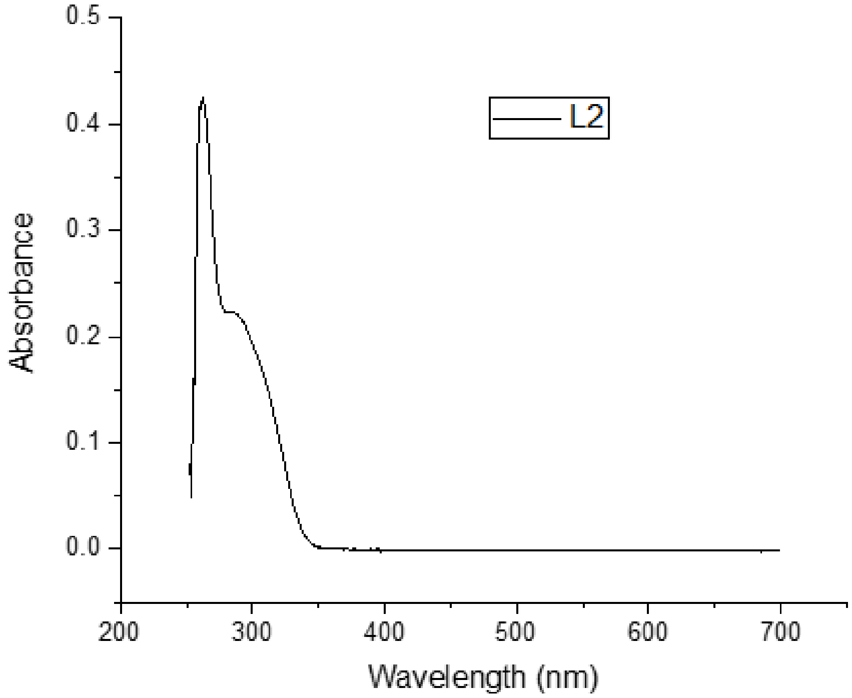
0.5
0.4
0.3
0.2
0.1
0.0
Absorbance
L2
200
300
400
500
600
700
Wavelength (nm)

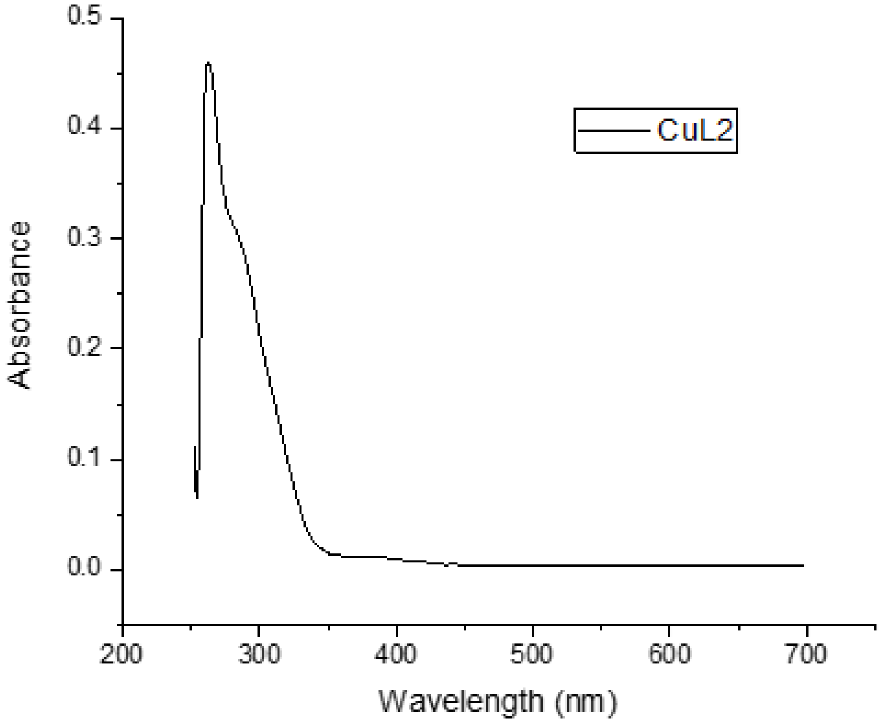
0.5
0.4
0.3
0.2
0.1
0.0
Absorbance
CuL2
200
300
400
500
600
700
Wavelength (nm)

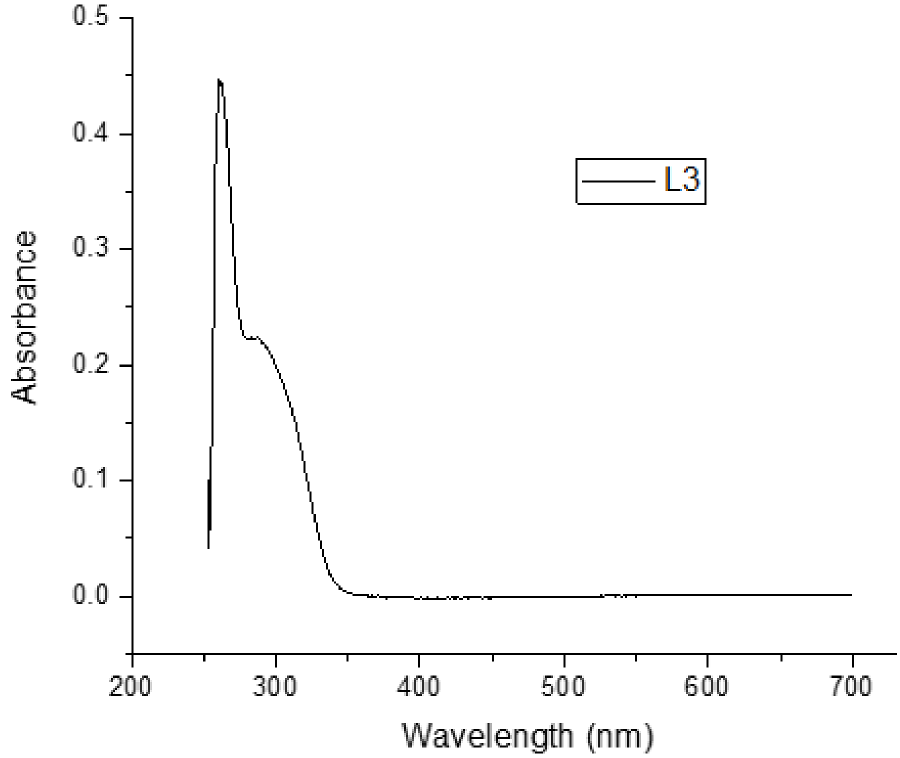
L3
Absorbance
Wavelength (nm)
0.5
0.4
0.3
0.2
0.1
0.0
200
300
400
500
600
700

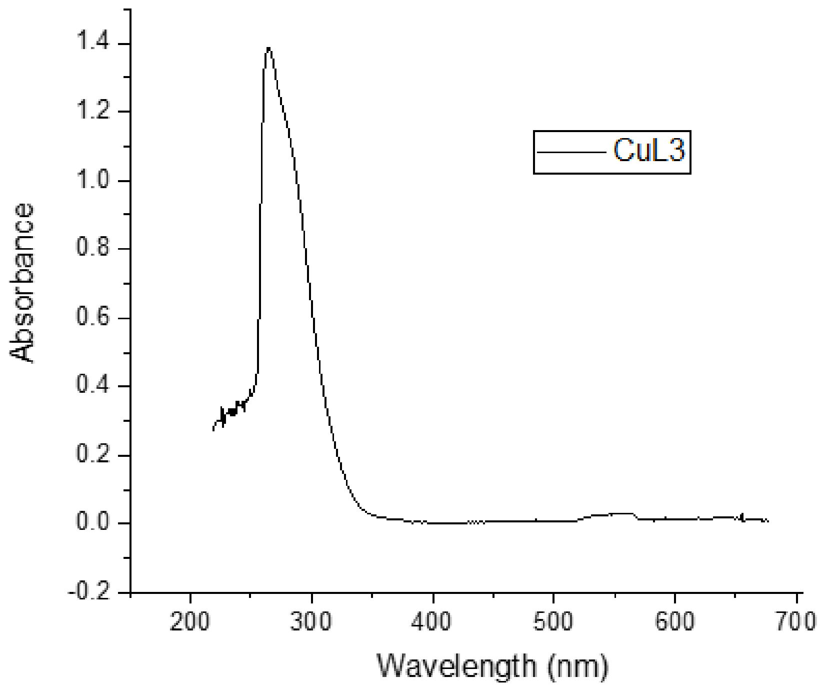
CuL3
Absorbance
Wavelength (nm)
1.4
1.2
1.0
0.8
0.6
0.4
0.2
0.0
-0.2
200
300
400
500
600
700

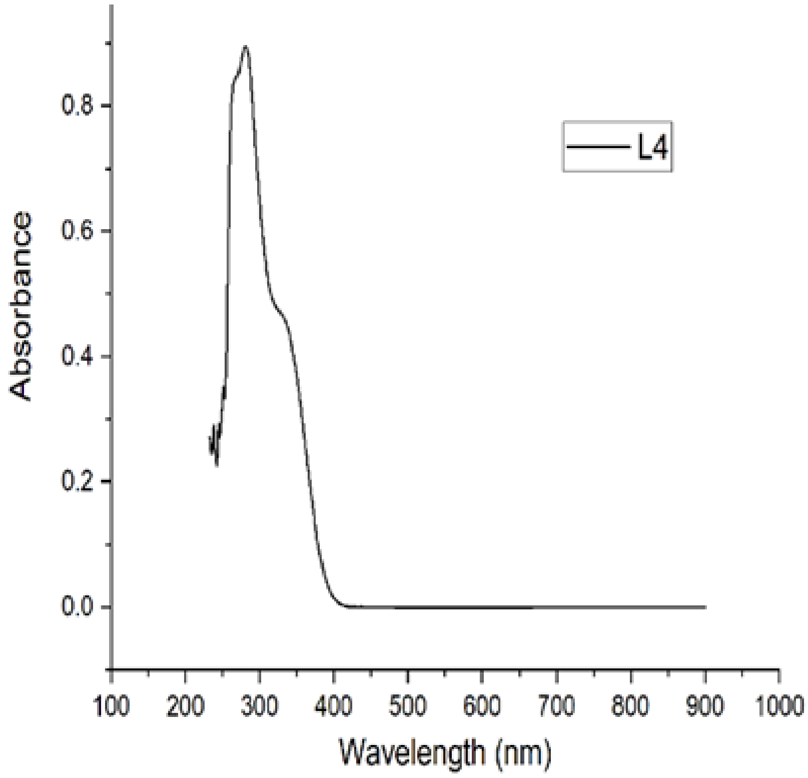
0.8
0.6
0.4
0.2
0.0
Absorbance
L4
100
200
300
400
500
600
700
800
900
1000
Wavelength (nm)

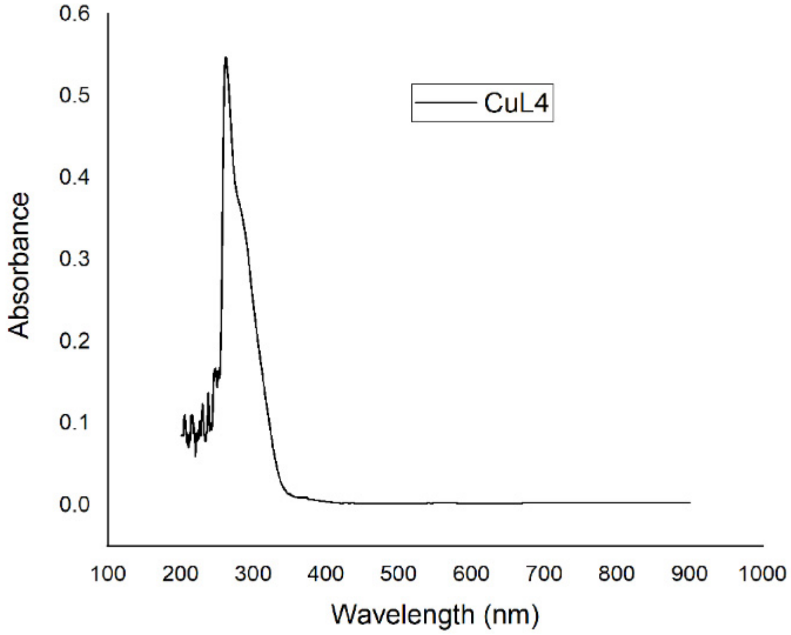
0.6
0.5
0.4
0.3
0.2
0.1
0.0
Absorbance
CuL4
100
200
300
400
500
600
700
800
900
1000
Wavelength (nm)

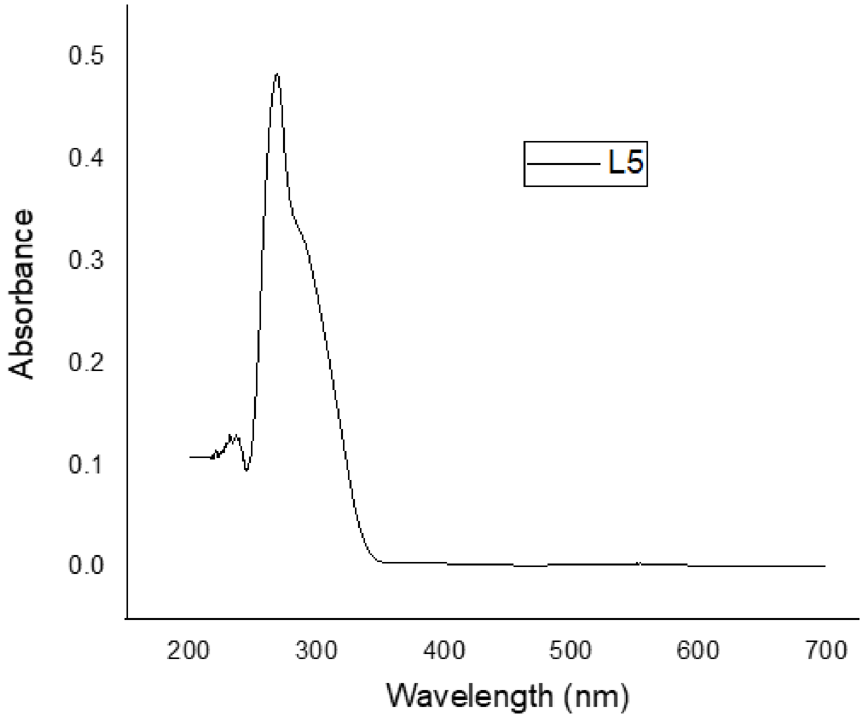

Absorbance
0.5
0.4
0.3
0.2
0.1
0.0
L5
200
300
400
500
600
700
Wavelength (nm)

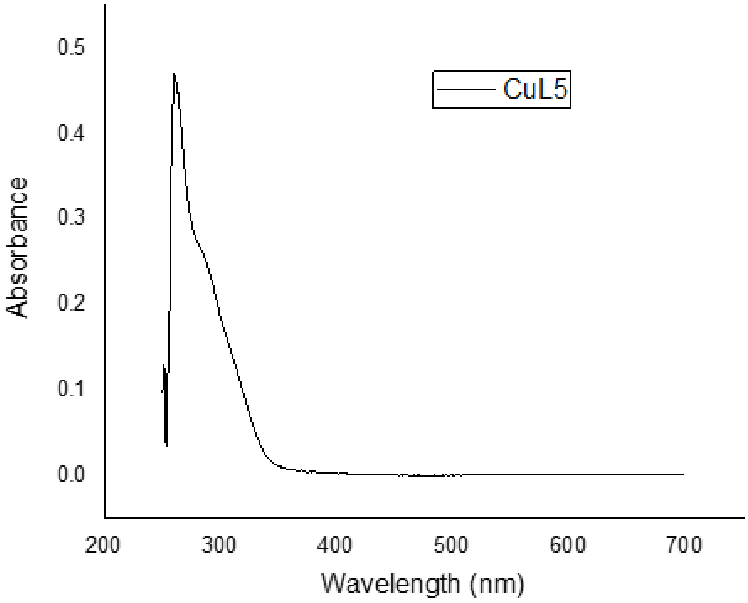

Absorbance
0.5
0.4
0.3
0.2
0.1
0.0
CuL5
200
300
400
500
600
700
Wavelength (nm)

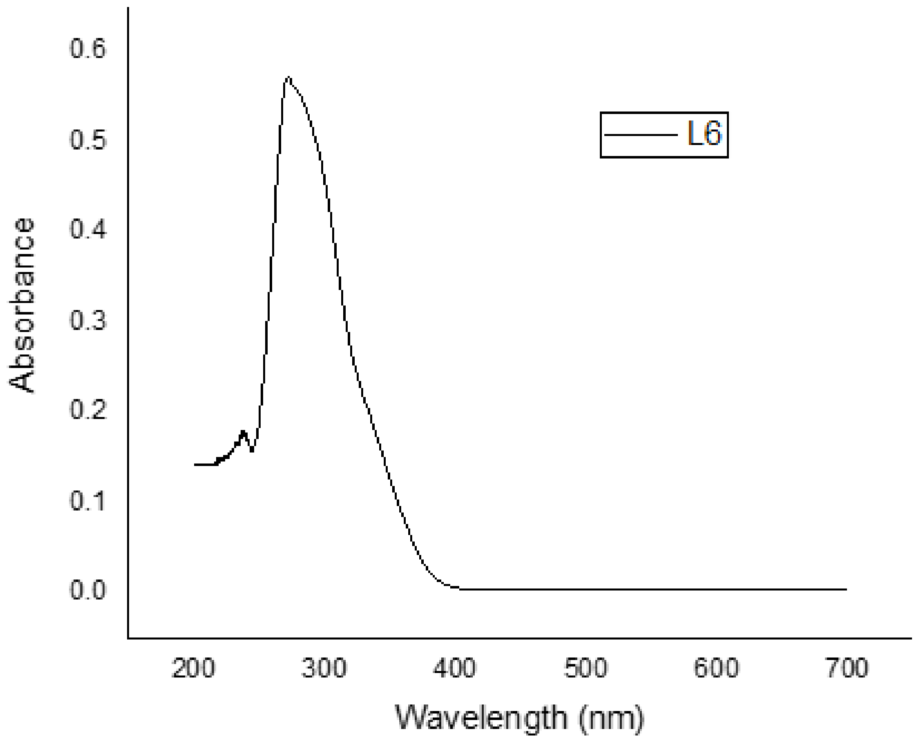
L6
Absorbance
0.6
0.5
0.4
0.3
0.2
0.1
0.0
200
300
400
500
600
700
Wavelength (nm)

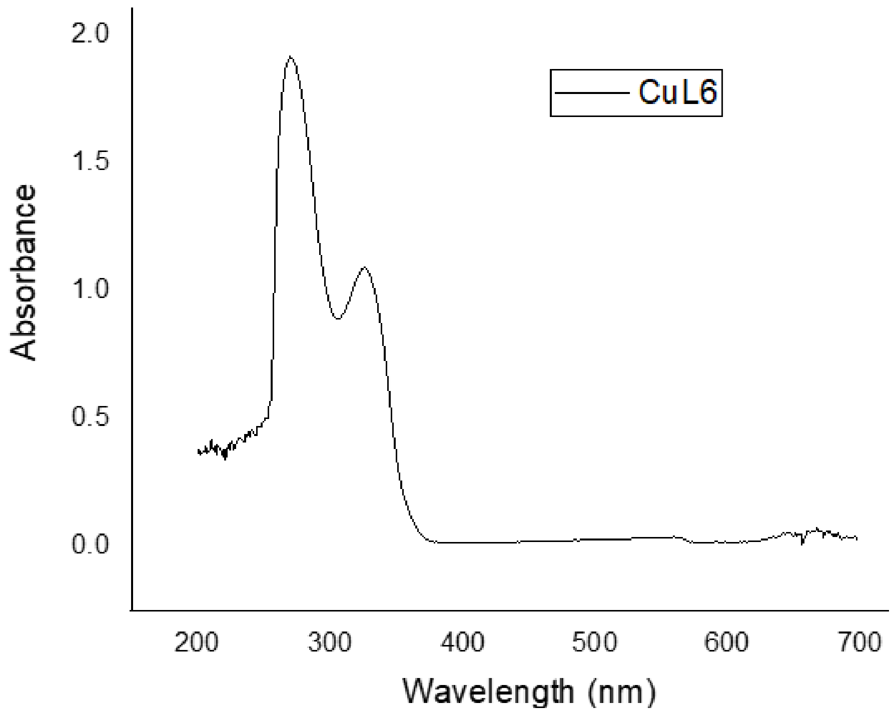
CuL6
Absorbance
2.0
1.5
1.0
0.5
0.0
200
300
400
500
600
700
Wavelength (nm)

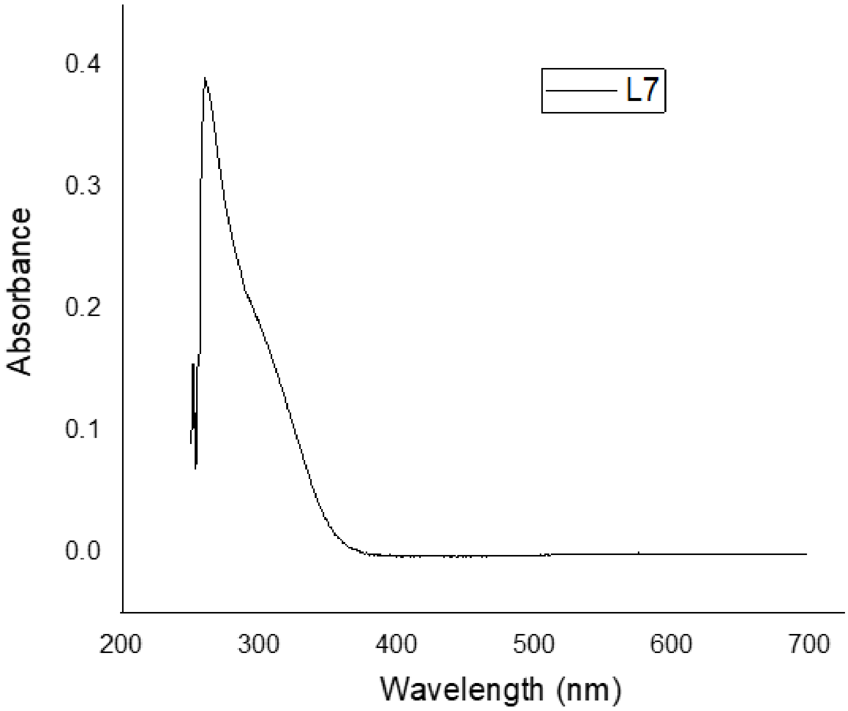
L7
Absorbance
0.4
0.3
0.2
0.1
0.0
200
300
400
500
600
700
Wavelength (nm)

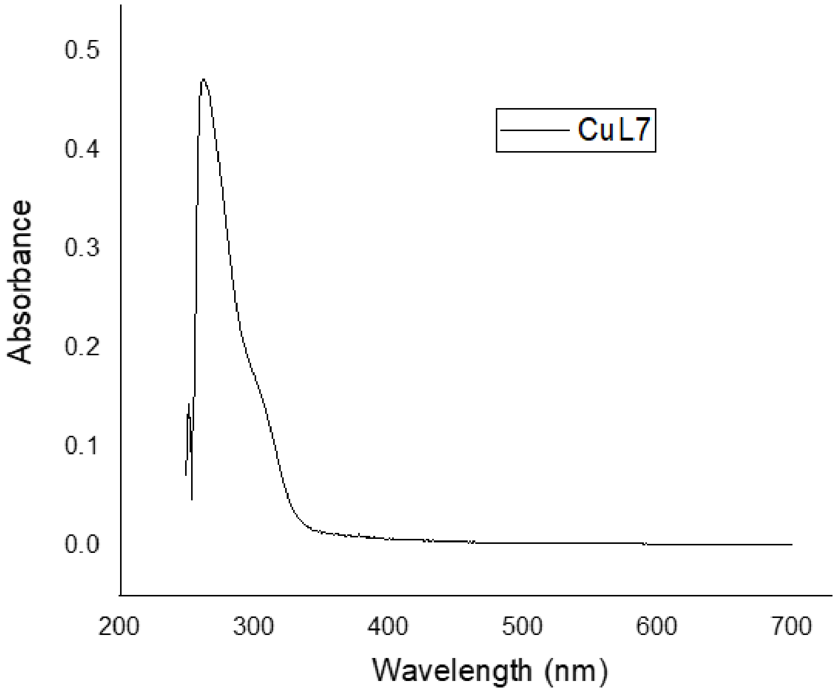
CuL7
Absorbance
0.5
0.4
0.3
0.2
0.1
0.0
200
300
400
500
600
700
Wavelength (nm)

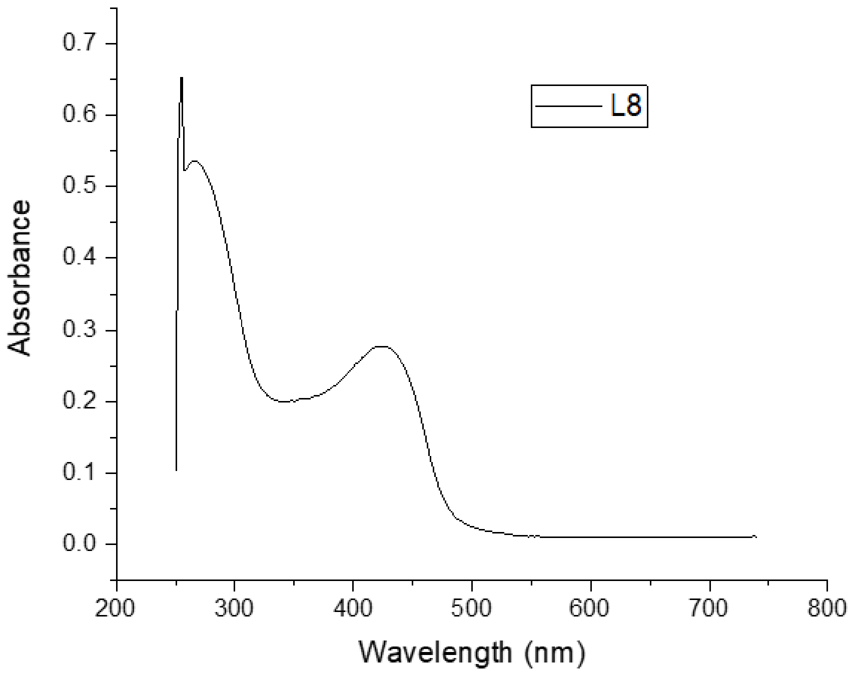
L8
Absorbance
0.7
0.6
0.5
0.4
0.3
0.2
0.1
0.0
200
300
400
500
600
700
800
Wavelength (nm)

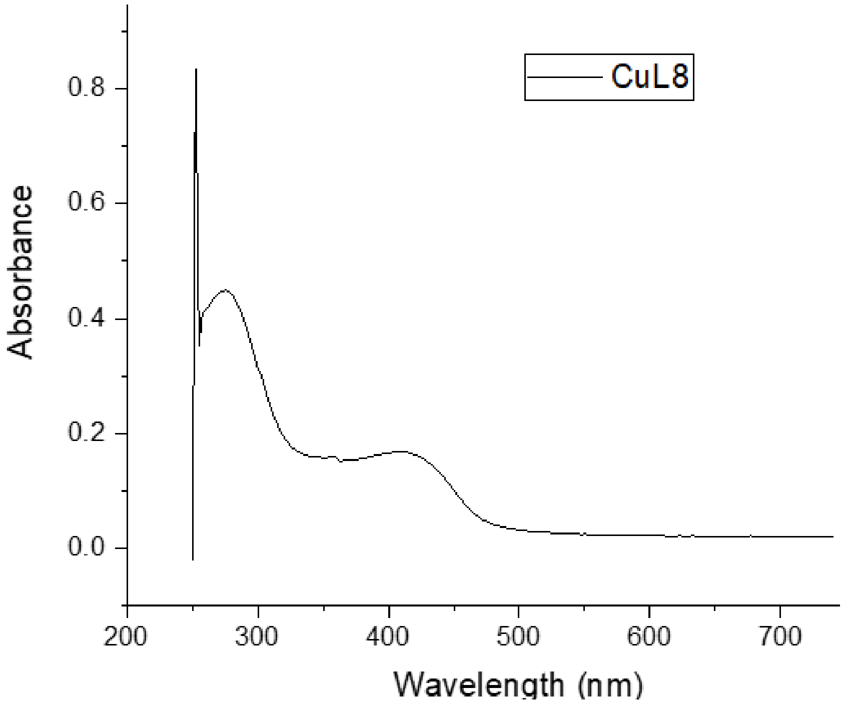
CuL8
Absorbance
0.8
0.6
0.4
0.2
0.0
200
300
400
500
600
700
Wavelength (nm)

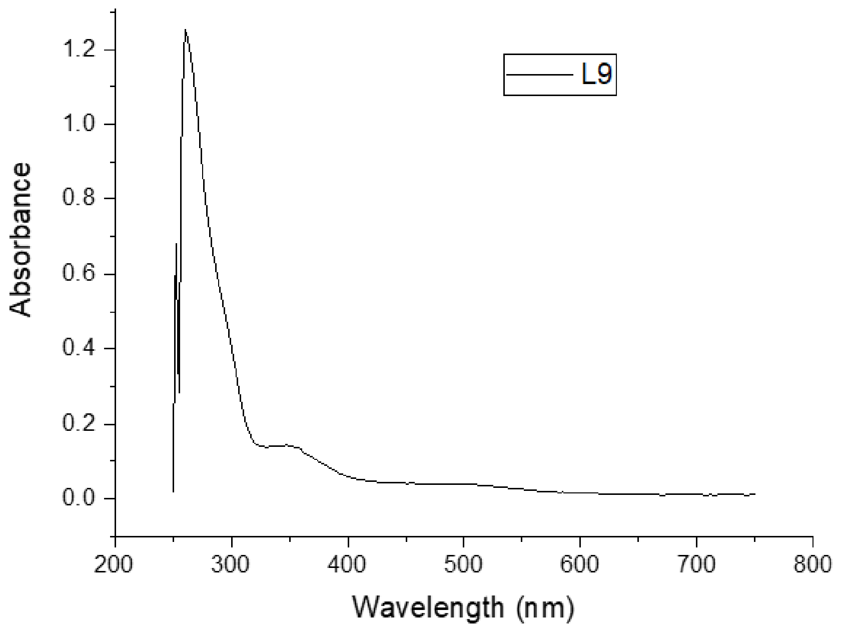
L9
Absorbance
1.2
1.0
0.8
0.6
0.4
0.2
0.0
200
300
400
500
600
700
800
Wavelength (nm)

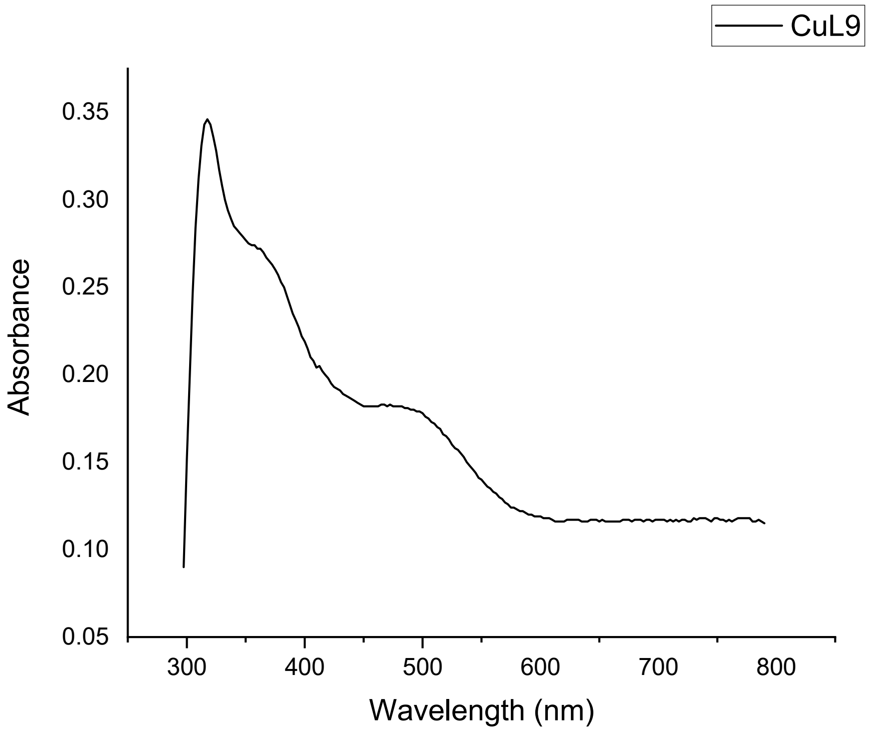
CuL9
Absorbance
0.35
0.30
0.25
0.20
0.15
0.10
0.05
300
400
500
600
700
800
Wavelength (nm)

^{1}H & ^{13}C NMR spectra of organic compounds

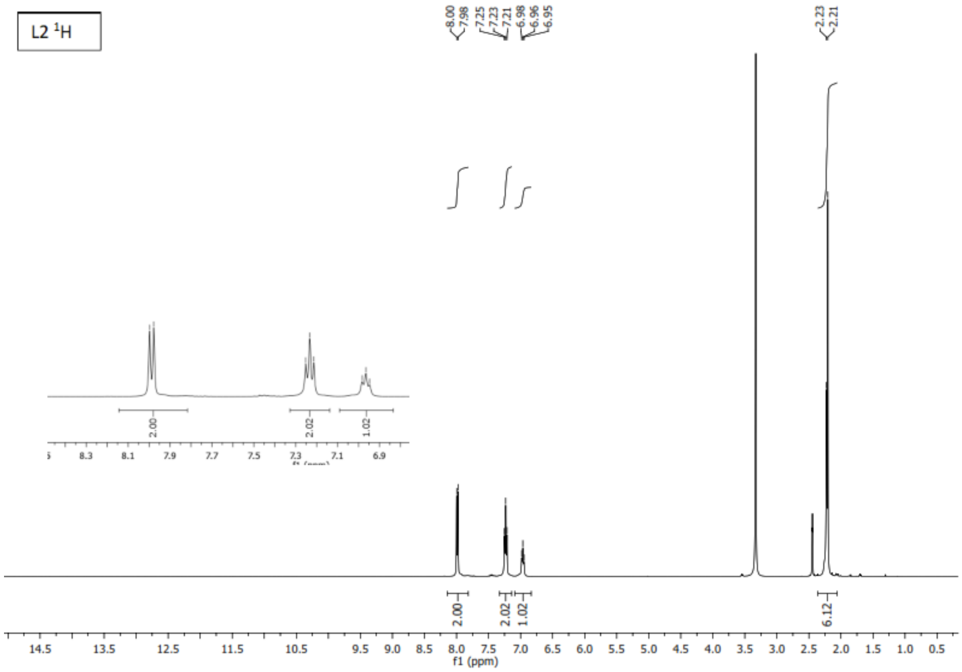

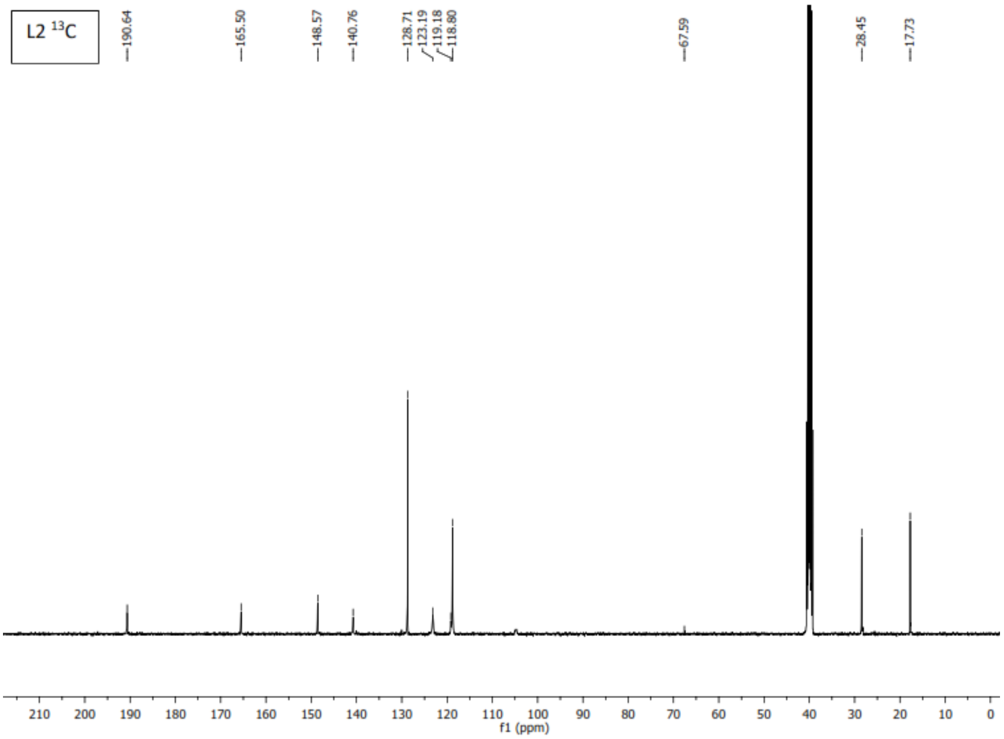

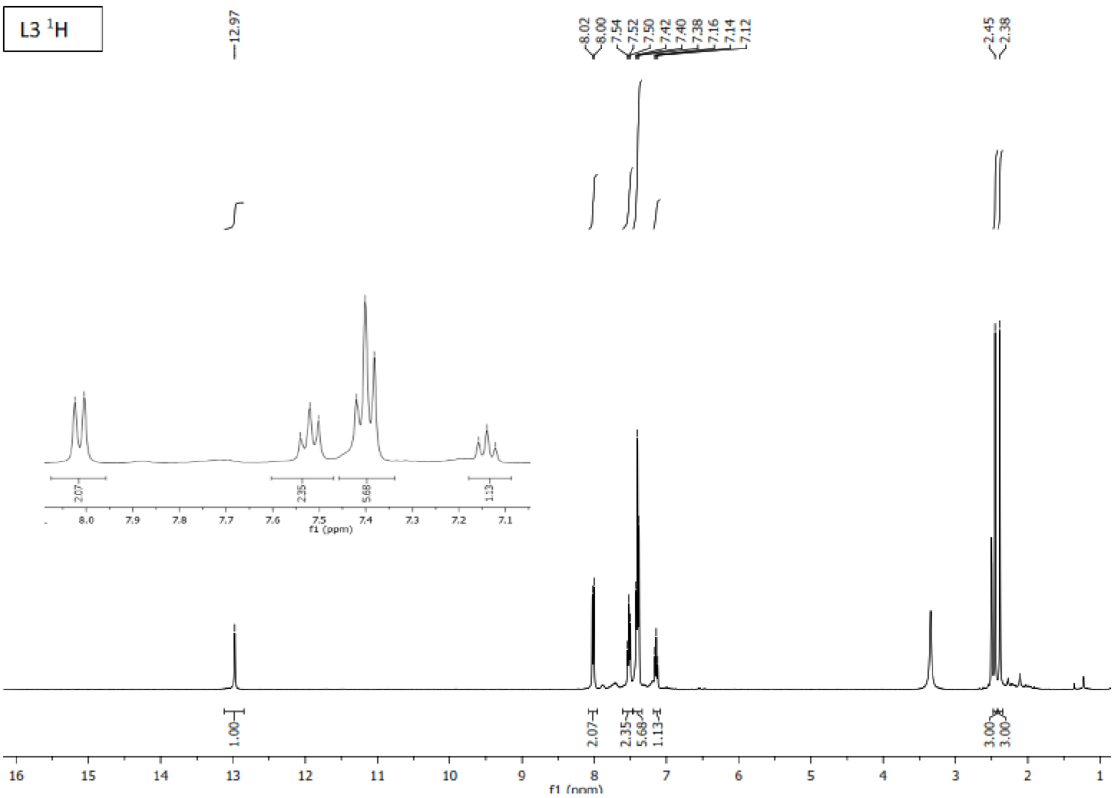
L3 1H
12.97
8.02
8.00
7.54
7.52
7.50
7.42
7.40
7.38
7.16
7.14
7.12
2.45
2.38
1.00
2.07
2.35
5.68
1.13
3.00
3.00
f1 (ppm)

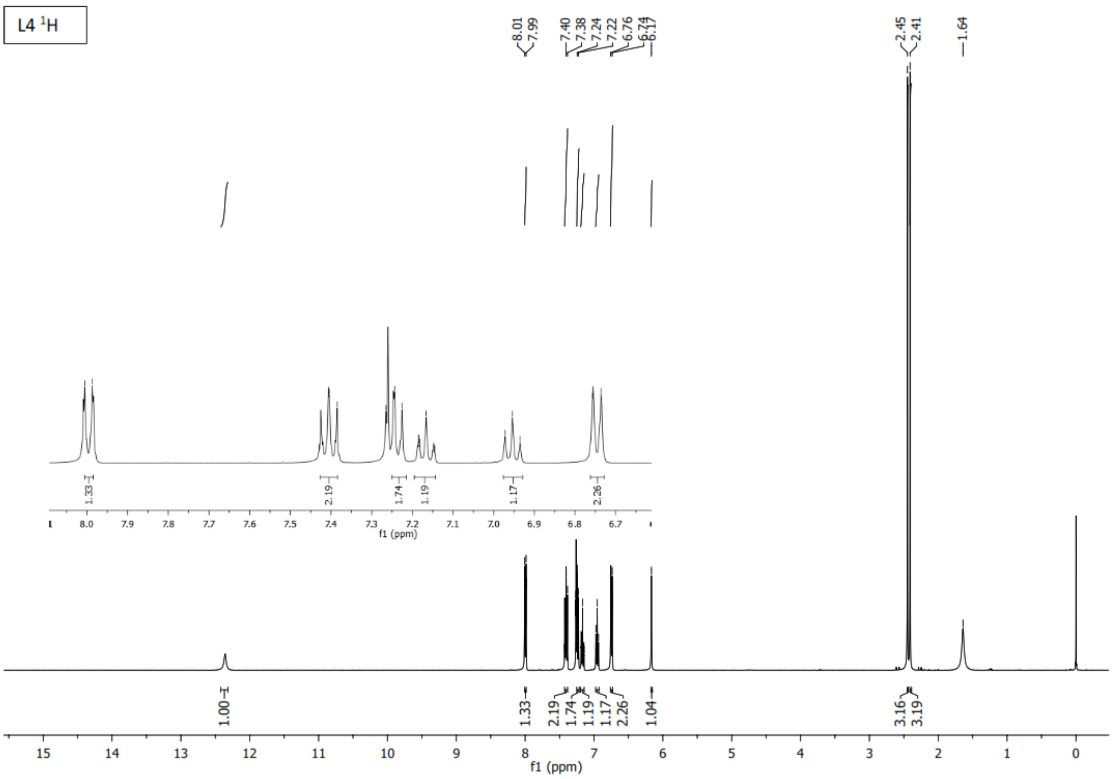
L4 1H
8.01
7.99
7.40
7.38
7.24
7.22
6.76
6.74
6.17
2.45
2.41
1.64
1.00
1.33
2.19
1.74
1.19
1.17
2.26
1.04
3.16
3.19
f1 (ppm)

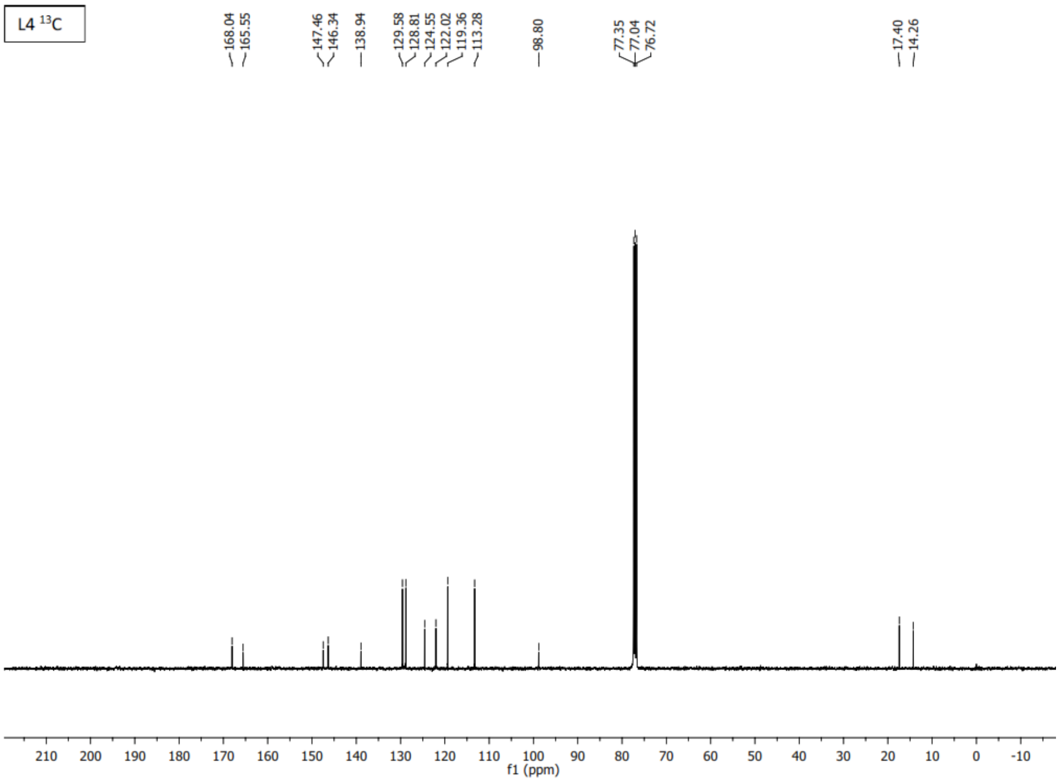
L4 13C
168.04
165.55
147.46
146.34
138.94
129.58
128.81
124.55
122.02
119.36
113.28
98.80
77.35
77.04
76.72
17.40
14.26
210 200 190 180 170 160 150 140 130 120 110 100 90 80 70 60 50 40 30 20 10 0 -10
f1 (ppm)

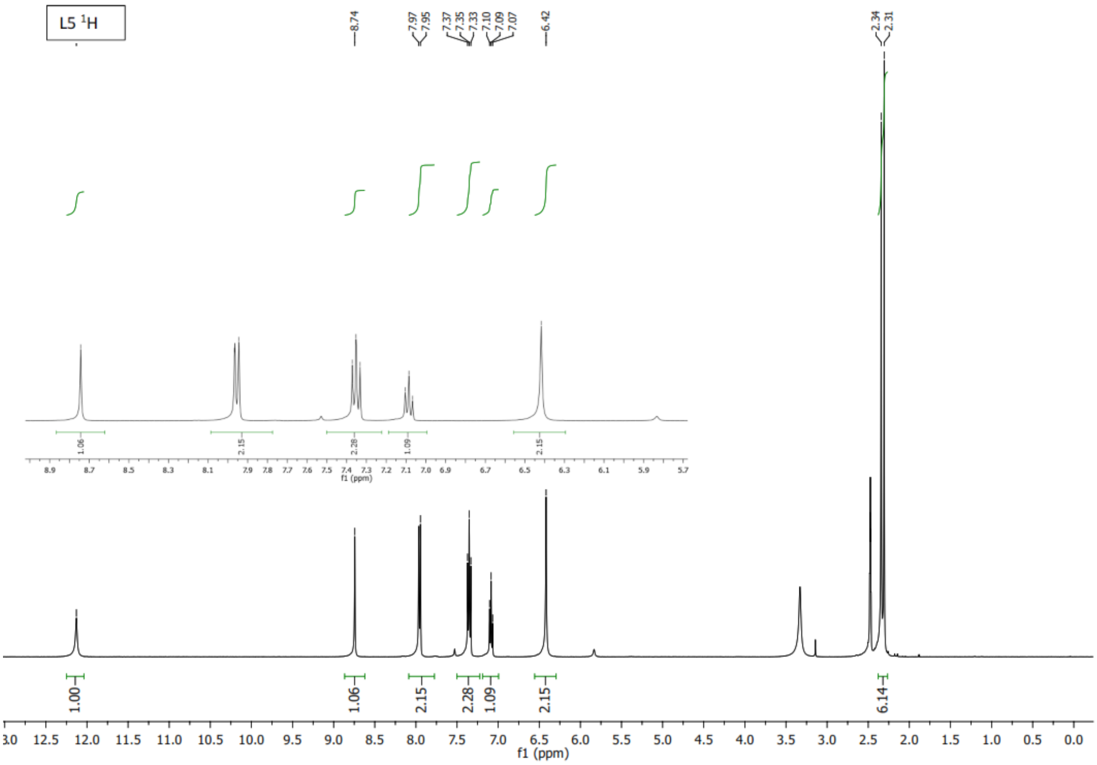
L5 1H
8.74
7.97
7.95
7.37
7.35
7.33
7.10
7.09
7.07
6.42
2.34
2.31
1.00
1.06
2.15
2.28
1.09
2.15
6.14
13.0 12.5 12.0 11.5 11.0 10.5 10.0 9.5 9.0 8.5 8.0 7.5 7.0 6.5 6.0 5.5 5.0 4.5 4.0 3.5 3.0 2.5 2.0 1.5 1.0 0.5 0.0
f1 (ppm)

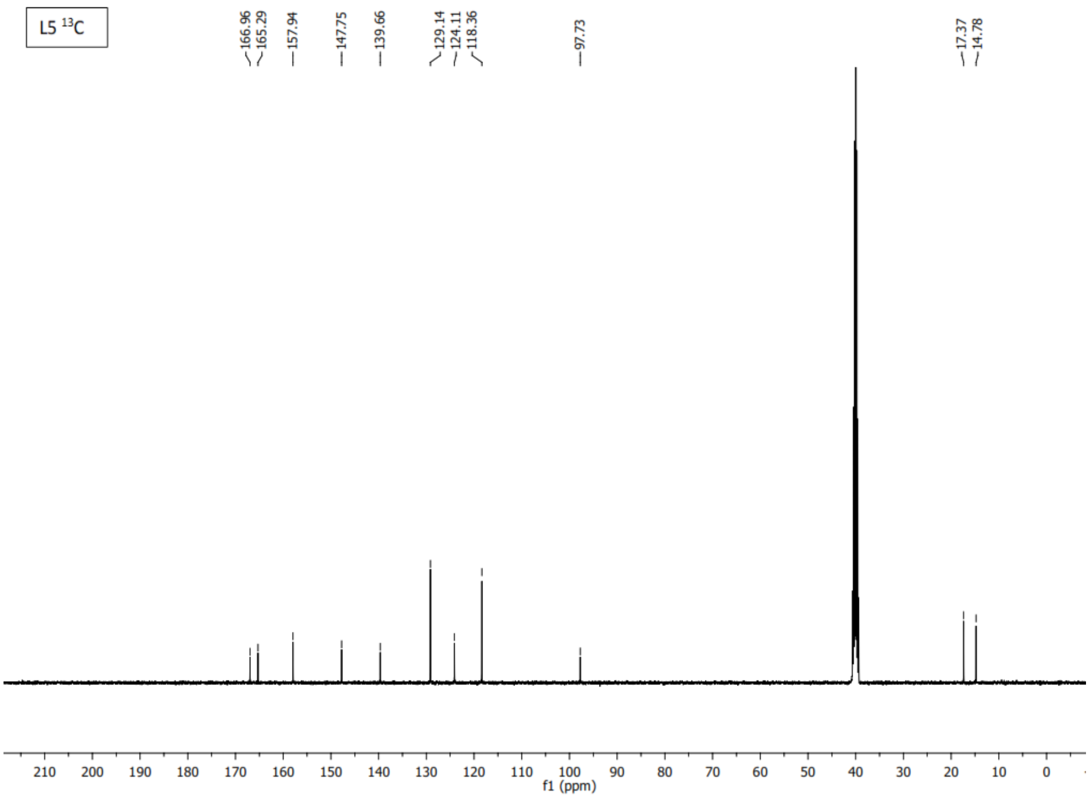
L5 13C
166.96
165.29
157.94
147.75
139.66
129.14
124.11
118.36
97.73
17.37
14.78
210 200 190 180 170 160 150 140 130 120 110 100 90 80 70 60 50 40 30 20 10 0
f1 (ppm)

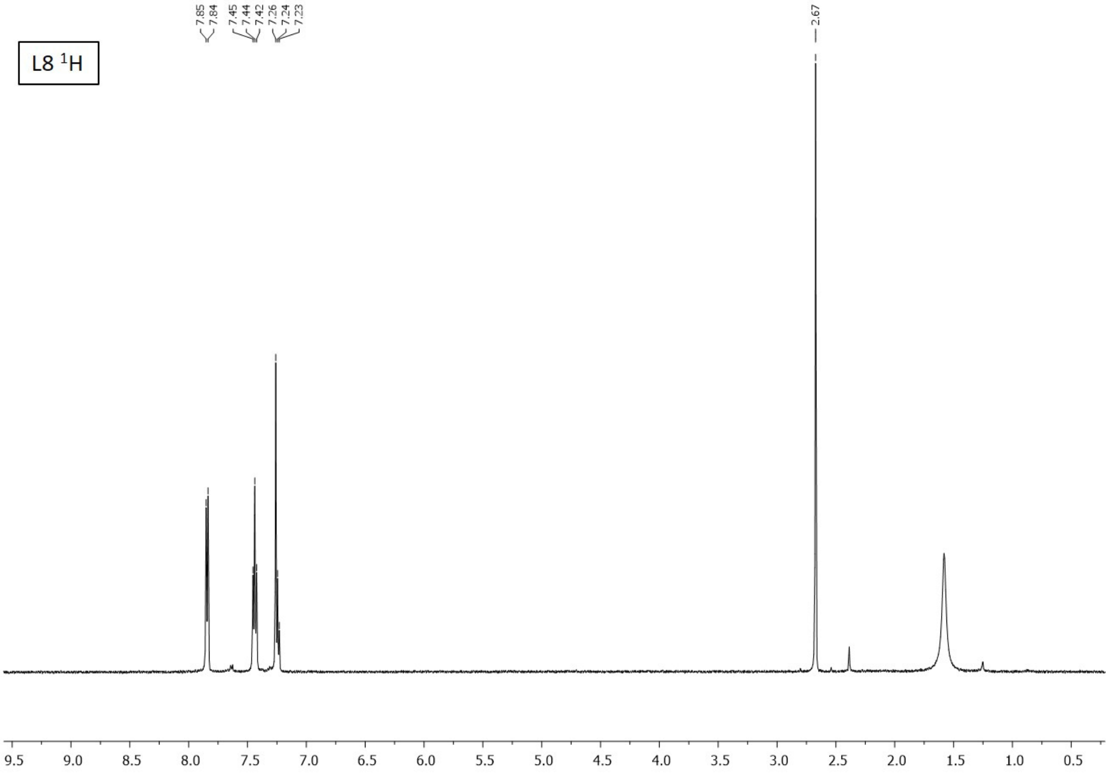
L8 1H
7.85
7.84
7.45
7.44
7.42
7.26
7.24
7.23
2.67
9.5 9.0 8.5 8.0 7.5 7.0 6.5 6.0 5.5 5.0 4.5 4.0 3.5 3.0 2.5 2.0 1.5 1.0 0.5

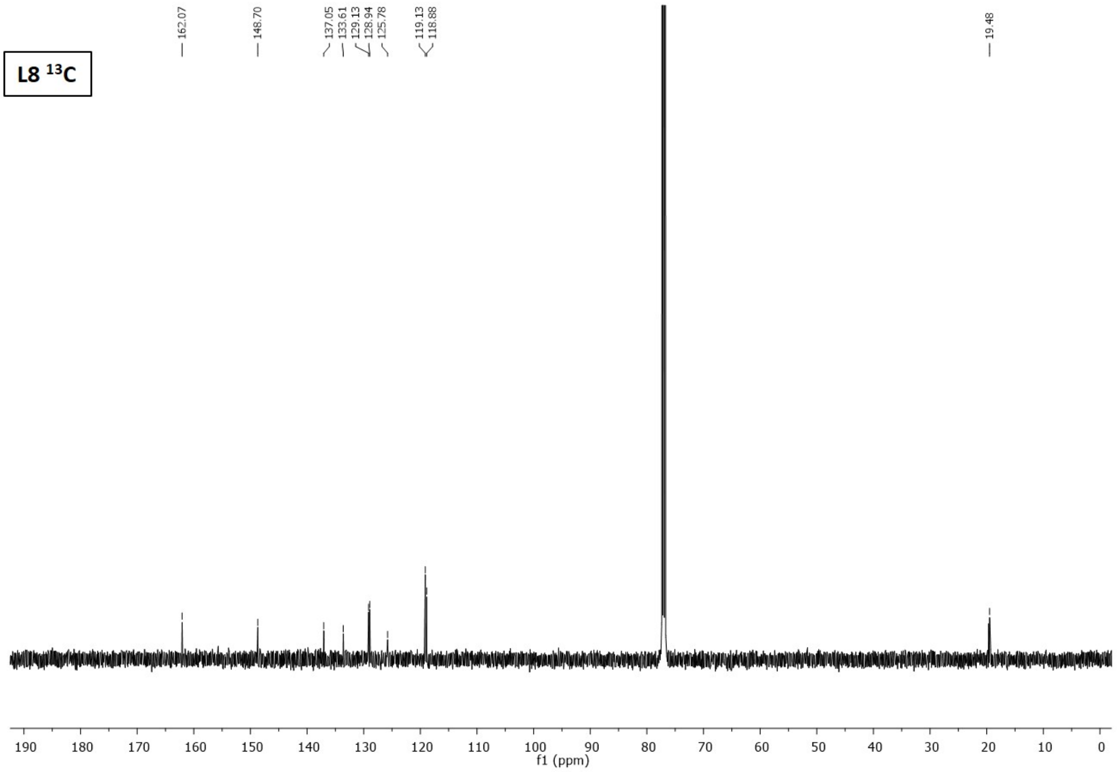
L8 13C
162.07
148.70
137.05
133.61
129.13
128.94
125.78
119.13
118.88
19.48
190 180 170 160 150 140 130 120 110 100 90 80 70 60 50 40 30 20 10 0
f1 (ppm)

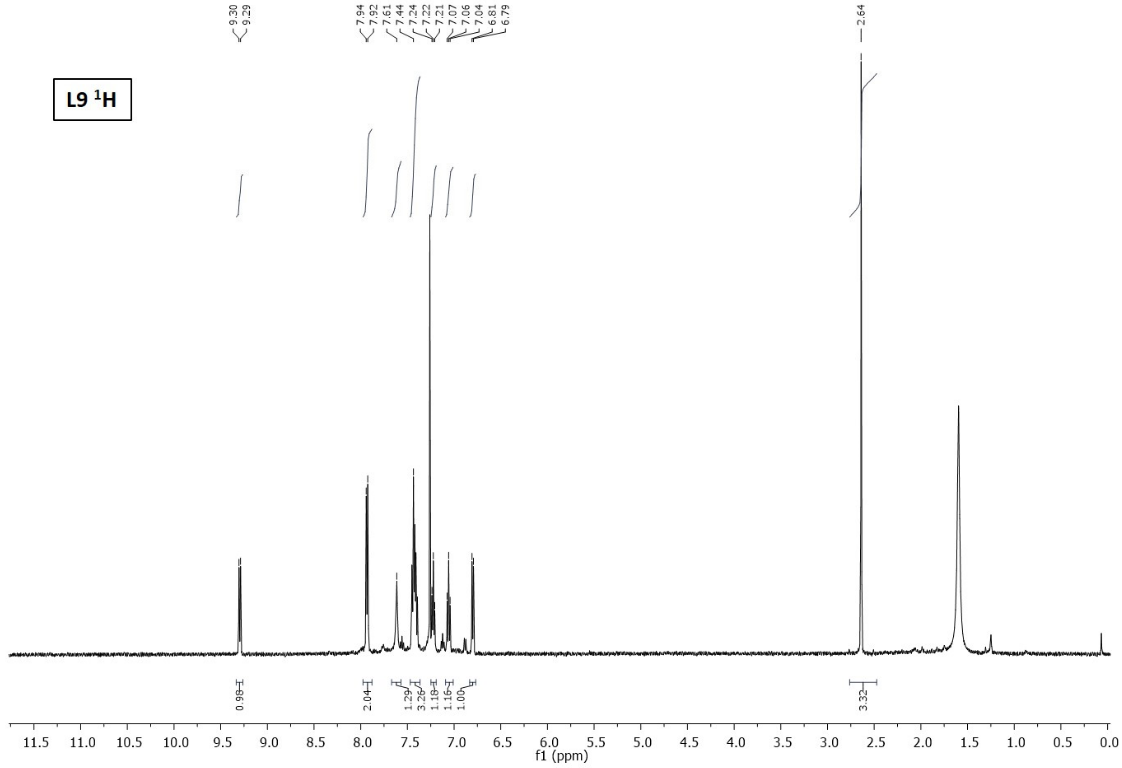
L9 1H
9.30
9.29
7.94
7.92
7.61
7.44
7.24
7.22
7.21
7.07
7.06
7.04
6.81
6.79
2.64
0.98
2.04
1.29
3.26
1.18
1.16
1.00
3.32
11.5 11.0 10.5 10.0 9.5 9.0 8.5 8.0 7.5 7.0 6.5 6.0 5.5 5.0 4.5 4.0 3.5 3.0 2.5 2.0 1.5 1.0 0.5 0.0
f1 (ppm)

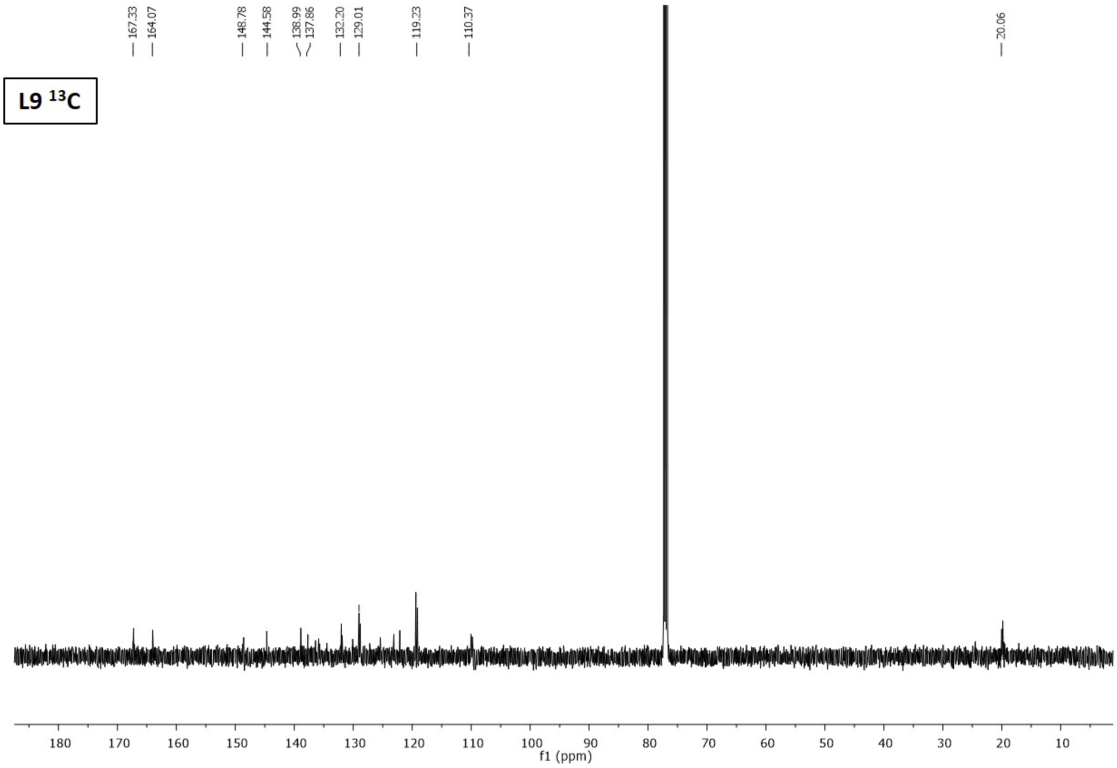

L9 13C
167.33
164.07
148.78
144.58
138.99
137.86
132.20
129.01
119.23
110.37
20.06
180 170 160 150 140 130 120 110 100 90 80 70 60 50 40 30 20 10
f1 (ppm)

<u>**Mass spectra of the organic compounds**</u>

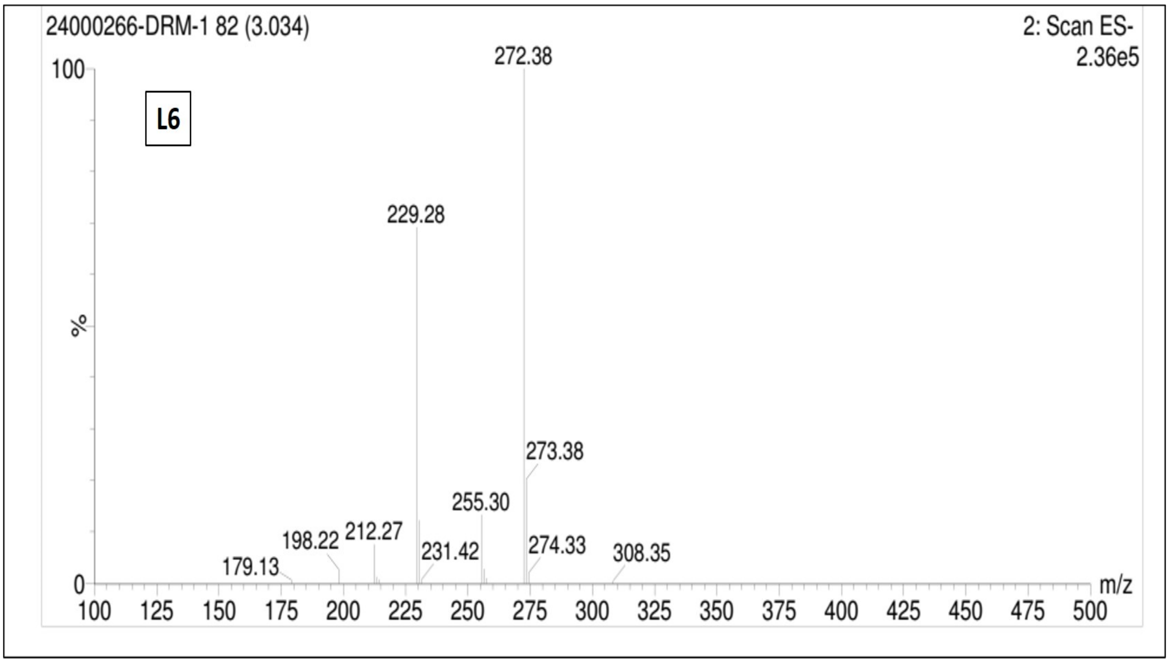

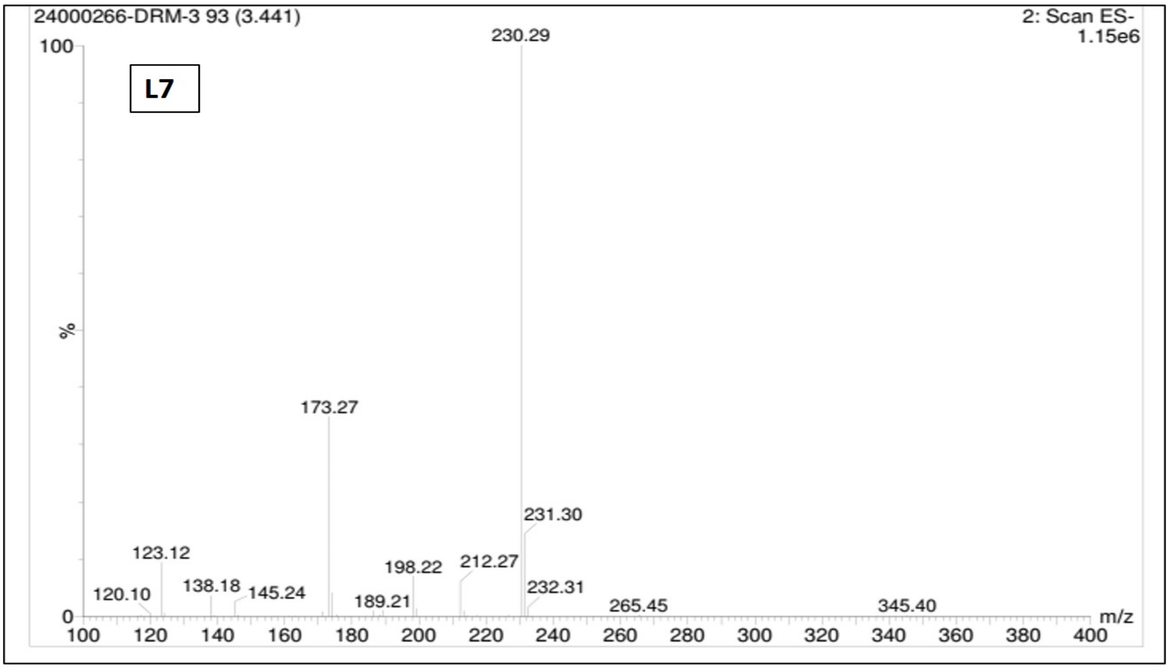

Table S1. Antioxidant activity results (HPSA, DPPH, ABTS) of edaravone derivatives (**L1**-**L9**) and their Cu(II)-complexes (**C1**-**C9**). Results are presented as IC_{50} ± SD/μg.mL^{-1}.

Compound	*IC_{50} ± SD/ μg.mL^{-1}*		
	**HPSA**	**DPPH**	**ABTS**
L1	100.56 ± 10.51	14.94 ± 0.31	51.88 ± 0.54
L2	115.67 ± 5.01	104.91 ± 1.59	270.26 ± 15.26
L3	73.52 ± 1.74	159.9 ± 1.20	483.78 ± 6.50
L4	73.29 ± 7.12	5.84 ± 0.20	42.26 ± 2.74
L5	75.37 ± 2.57	114.55 ± 1.04	283.72 ± 9.47
L6	7.61 ± 0.31	2.94 ± 0.04	38.70 ± 1.63
L7	122.59 ± 1.75	4.77 ± 0.07	45.53 ± 1.25
L8	21.61 ± 0.86	26.83 ± 0.86	110.11 ± 1.70
L9	86.03 ± 1.55	90.60 ± 0.63	101.16 ± 2.25
C1	178.73 ± 5.08	62.38 ± 1.11	86.62 ± 4.90
C2	232.10 ± 12.07	75.47 ± 0.73	157.34 ± 1.81
C3	108.98 ± 6.65	40.82 ± 0.79	59.11 ± 0.54
C4	152.87 ± 8.27	66.65 ± 0.76	122.33 ± 1.18
C5	231.86 ± 16.18	89.99 ± 1.50	131.59 ± 2.27
C6	188.77 ± 13.61	36.35 ± 0.86	41.27 ± 2.18
C7	207.52 ± 13.48	14.15 ± 0.50	18.23 ± 0.78
C8	166.79 ± 9.95	22.55 ± 0.92	40.55 ± 1.31
C9	122.96 ± 7.62	41.21 ± 0.93	34.12 ± 2.49
